# The m^6^A reader YTHDC2 inhibits lung adenocarcinoma tumorigenesis by suppressing SLC7A11-dependent antioxidant function

**DOI:** 10.1016/j.redox.2020.101801

**Published:** 2020-11-18

**Authors:** Lifang Ma, Tianxiang Chen, Xiao Zhang, Yayou Miao, Xiaoting Tian, Keke Yu, Xin Xu, Yongjie Niu, Susu Guo, Congcong Zhang, Shiyu Qiu, Yongxia Qiao, Wentao Fang, Lutao Du, Yongchun Yu, Jiayi Wang

**Affiliations:** a Department of Thoracic Surgery, Shanghai Chest Hospital, Shanghai Jiao Tong University, Shanghai, 200030, China; b Shanghai Institute of Thoracic Oncology, Shanghai Chest Hospital, Shanghai Jiao Tong University, Shanghai, 200030, China; c Shanghai Lung Cancer Center, Shanghai Chest Hospital, Shanghai Jiao Tong University, Shanghai, 200030, China; d Department of Bio-bank, Shanghai Chest Hospital, Shanghai Jiao Tong University, Shanghai, 200030, China; e Shanghai Municipal Hospital of Traditional Chinese Medicine, Shanghai University of Traditional Chinese Medicine, Shanghai, 200071, China; f Department of Clinical Laboratory Medicine, Shanghai Tenth People's Hospital of Tongji University, Shanghai, 200072, China; g School of Public Health, Shanghai Jiao Tong University School of Medicine, Shanghai, 200025, China; h Department of Clinical Laboratory, The Second Hospital of Shandong University, Jinan, 250033, Shandong province, China

**Keywords:** System X_C_^−^, Cystine uptake, METTL3, Lipid peroxidation, m^6^A RNA methylation

## Abstract

The biological functions of N6-methyladenosine (m^6^A) RNA methylation are mainly dependent on the reader; however, its role in lung tumorigenesis remains unclear. Here, we have demonstrated that the m^6^A reader YT521-B homology domain containing 2 (YTHDC2) is frequently suppressed in lung adenocarcinoma (LUAD). Downregulation of YTHDC2 was associated with poor clinical outcome of LUAD. YTHDC2 decreased tumorigenesis in a spontaneous LUAD mouse model. Moreover, YTHDC2 exhibited antitumor activity in human LUAD cells. Mechanistically, YTHDC2, via its m^6^A-recognizing YTH domain, suppressed cystine uptake and blocked the downstream antioxidant program. Administration of cystine downstream antioxidants to pulmonary YTHDC2-overexpressing mice rescued lung tumorigenesis. Furthermore, solute carrier 7A11 (SLC7A11), the catalytic subunit of system X_C_^−^, was identified to be the direct target of YTHDC2. YTHDC2 destabilized *SLC7A1*1 mRNA in an m^6^A-dependent manner because YTHDC2 preferentially bound to m^6^A-modified *SLC7A1*1 mRNA and thereafter promoted its decay. Clinically, a large proportion of acinar LUAD subtype cases exhibited simultaneous YTHDC2 downregulation and SLC7A11 elevation. Patient-derived xenograft (PDX) mouse models generated from acinar LUAD showed sensitivity to system X_C_^−^ inhibitors. Collectively, the promotion of cystine uptake via the suppression of YTHDC2 is critical for LUAD tumorigenesis, and blocking this process may benefit future treatment.

## Highlights

• The m^6^A reader YTHDC2 is frequently suppressed in LUAD and indicates poor prognosis.
• YTHDC2 suppresses the antioxidant function of system X_C_^−^ via its m^6^A reading domain.
• The mRNA encoding SLC7A11 is a direct target of YTHDC2.
• YTHDC2 preferentially accelerates the decay of m^6^A-methylated *SLC7A1*1 mRNA.
• LUAD with YTHDC2 suppression is sensitive to system X_C_^−^ inhibitors.

## Graphical abstract

**Figure:**
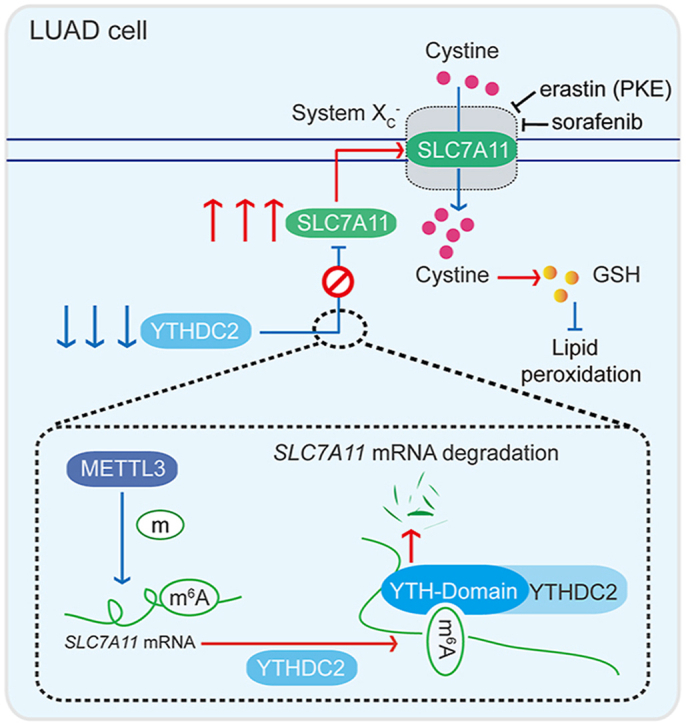

## 1 Introduction

Lung adenocarcinoma (LUAD) is the most prevalent type of non-small cell lung cancer [1]. However, the mechanisms underlying LUAD tumorigenesis are largely unknown. Therefore, it is urgent to further explore the molecular pathogenesis of LUAD to develop novel treatment strategies and reduce mortality.

Numerous studies have demonstrated the crucial role of N6-methyladenosine (m^6^A) RNA methylation in various human cancers, including lung cancer [[2], [3], [4], [5]]. m^6^A RNA methylation is the most common internal posttranscriptional methylation of mRNAs. It is a reversible process controlled by the m^6^A WER system, comprised of writers (W), erasers (E), and readers (R). The major m^6^A writer is a methyltransferase complex, while the eraser is an enzyme that catalyzes demethylation. In addition to the biological effect on target transcripts, the final outcome of m^6^A methylation is determined on the destiny after recognition and binding by the specific reader. Although the function of several m^6^A readers has been established in liver, colorectal, and ovarian cancer [4,6,7], to the best of our knowledge, the role of readers in LUAD is largely unknown. YTHDC2 functions as a reader to regulate spermatogenesis and suppress liver steatosis [[8], [9], [10]]. YTHDC2 is believed to be the final member of the YTH protein family. However, whether and how YTHDC2 exerts its function in LUAD has not yet been investigated.

Although m^6^A methylation is known to be an important mechanism involved in cell proliferation, autophagy and apoptosis [[11], [12], [13]], its role in metabolic processes is still not known. Small molecules, such as erastin and sorafenib, can trigger lipid peroxidation by suppressing system X_C_^−^, a cystine/glutamate antiporter [[14], [15], [16]]. System X_C_^−^ maintains redox homeostasis through the import of cystine, an essential precursor for glutathione (GSH) biosynthesis. Inhibiting system X_C_^−^ impairs tumor growth and induces cell death [16,17]. System X_C_^−^ is a heterodimer that consists of two core components: SLC7A11 and solute carrier 3A2 (SCL3A2). Previous studies have revealed that upregulation of SLC7A11 is negatively correlated with lipid peroxidation [14]. SLC7A11 can be regulated at the transcriptional and translational levels [16,18,19]. However, whether SLC7A11 is modulated at the m^6^A level in LUAD remains elusive thus far.

In this study, we found that YTHDC2 inhibits LUAD tumorigenesis in vivo and in vitro and is largely dependent on its YTH domain. Furthermore, *SLC7A1*1 mRNA was modified by m^6^A and bound by YTHDC2. YTHDC2 promotes *SLC7A1*1 mRNA degradation in an m^6^A-dependent manner. Using cell-based experiments, mouse models, and clinical LUAD specimens (total 382), our data demonstrate the critical tumor suppressor roles of the m^6^A reader YTHDC2 in LUAD tumorigenesis.

## 2 Materials and methods

### 2.1 Clinical specimen

Tissues were obtained from 382 LUAD patients who had received curative surgery at Shanghai Chest Hospital and the Second Hospital of Shandong University. For cohort#1, 192 LUAD patients were recruited from January 2018 to March 2019; for cohort#2, 100 LUAD patients with acinar predominant subtype were recruited from December 2013 to December 2014; for cohort#3, 30 LUAD patients were recruited from north China; and for cohort#4, 60 LUAD patients were recruited from March 2017 to June 2017. Written informed consents were obtained from each enrolled patient, according to the guidelines of the declaration of Helsinki. This study was approved by Institutional Ethics Committee of Shanghai Chest Hospital.

### 2.2 Bioinformatics

We used GEPIA, UALCAN, TCGA and Oncomine database to analyze the transcriptional level of genes. The GEPIA and the Kaplan-Meier plotter databases were used to analyze survival information. The correlation between YTHDC2 and SLC7A11 was evaluated in GEPIA database.

### 2.3 Animal experiments

*Kras*^*LSL-G12D/+*^*; p53*^*LSL-R172H/+*^ (KP) strains were purchased from the Jackson Laboratory. The KP mice were intranasal administered with 25 μl adeno-associated type 5 virus (AAV5) particles (2 × 10^12^ viral particles/ml) encoding for Cre recombinase (Cre), thereby initiated lung tumorigenesis. The mice were also simultaneously intranasal infected with AAV5 encoding YTHDC2^WT^ (WT indicates wide-type of YTHDC2) or YTHDC2 ^ΔYTH^ (ΔYTH indicates YTH domain dele of YTHDC2) under anaesthesia. Meanwhile, the empty-AAV5 was parallel infected and treated as a negative control. Thereby, the KP-based pulmonary expressing YTHDC2^WT^ (KPY^WT^), YTHDC2 ^ΔYTH^ (KPY^ΔYTH^) and control KPE mice were generated. Afterwards, two mice/group were sacrificed weekly to monitor the incidence of lung tumor from 6 to 12 wk after initiation of tumorigenesis. For drug assessment, vehicle, N-acetyl-cysteine (NAC, 100 mg/kg/day, Sigma, #A7250, St Louis, MO, USA) or GSH (100 mg/kg/day, Solarbio, #G8180, Beijing, China) dissolved in distilled water were intraperitoneally injected into the KPE and KPY^WT^ mice for 4 weeks beginning at 10 wk post AAV5 infection (n = 8 mice/group). The survival rate was calculated by Kaplan-Meier method and statistical significance was assessed by log-rank tests.

For patient-derived xenograft (PDX) mice experiments, ~2–3 mm^3^ fresh tumor specimens from acinar LUAD were implanted into 4-6-wk-old athymic nude mice (Jiesijie, Shanghai, China). For drug assessment, DMSO, piperazine erastin (PKE, 20 mg/kg/day, MedChemExpress, MCE, #HY-100887, Monmouth, NJ, USA) or sorafenib (80 mg/kg/day, Selleck, #S7394, Houston, TX, USA) were subcutaneously injected into the third generation PDX mice at 10 days after implantation (n = 8 mice/group). The tumor volumes were measured every 10 days after drug treatment and the tumors were separated for further IHC, MDA and 4-HNE analysis after sacrificed.

For xenograft experiments, H1299 (1 × 10^7^) or H1975 cells (1.5 × 10^7^) with different treatment were subcutaneously injected into 4-6-week-old athymic nude mice (n = 8/group). Tumor weight and cystine uptake were assessed after sacrificing the mouse at day 40 after implantation. The tumor volume was calculated as 0.5 × L × W^2^, (L indicates length, while W indicates width). All mouse experiments were performed according to the institutional guidelines of Shanghai Chest Hospital.

### 2.4 Cell culture

HEK-293 T, human lung epithelial cell (hLepC) BEAS-2B, human LUAD cell lines A549, NCI-H1299, PC-9, NCI-H1975, NCI-H441, NCI-H1650, HCC827, NCI-H292 and Calu-1 were purchased from FuHeng Cell Center (Shanghai, China). Primary patient-derived priLow-YTHDC2 (pLY#1–8, YTHDC2 is expressed below the average level) and priHigh-YTHDC2 (pHY#1–8, YTHDC2 is expressed beyond the average level) LUAD cells were obtained from fresh LUAD tissues. All cell lines were cultured in DMEM (GIBCO) with 10% FBS (HyClone, Logan, UT, USA) and 1% penicillin/streptomycin (Invitrogen, Carlsbad, CA, USA).

For cells in Cultrex® Basement Membrane Extract (BME)-based 3D culture system, firstly, 50 μl/well BME was added into 96-well plate. Subsequently, cell suspension (8000–10,000 cell/200 μl/well) was seeded on the plate coated with BME. Culture medium was changed every 3 days. Spheroid number and size were calculated after 7 days culture.

### 2.5 Reagents and plasmids

Reagents used in cell-based experiment were: cycloheximide (CHX, 10 μg/ml, Sigma, #C7698), Actinomycin D (ActD, 5 μg/ml, MCE, #HY17559), C11-BODIPY^581/591^ (5 mM, Invitrogen, #D3861), DEPC water (Solarbio, #R1600) and RNasin (Solarbio, #R8060, 5U/μl). For crispr/cas9 knockout of YTHDC2, NRF2, XRN2 and EXOSC10, single guide RNAs (sgRNAs) were cloned into the LentiCrisprV2 plasmid (Addgene, Cambridge, MA, USA). Lentiviral-based plasmids expressing METTL3, ATF4, YTHDC2^WT^, YTHDC2 ^ΔYTH^, YTHDC2^sg2−resistant (res)^ (including the ones with or without an HA- or FLAG-tag), HA-tagged YTHDC2^ΔYTH (sg2−res)^, and SLC7A11 shRNA-expressing plasmid were purchased from GeneCopoeia Biotech Lnc (Rockville, MD, USA) and Zuorun Biotech Ltd (Shanghai, China), respectively. SLC7A11-expressing and METTL3 shRNA-expressing plasmids were purchased from Zuorun Biotech Ltd. YTHDC2^WT^-HA and YTHDC2 ^ΔYTH^-HA were also cloned into pCDNA3.1(+). The sequences for sgRNA and shRNA, and primers for cloning are listed in Supplementary Table S1.

### 2.6 Evaluation of gene expression

Immunoblotting (IB) was performed according to the conventional protocol, which is available elsewhere. The primary antibodies used for IB were: anti-YTHDC2 (Abcam, #ab176846, Cambridge, MA, USA), anti-SLC7A11 (#ab175186), anti-ATF4 (#ab184909), anti-NRF2 (#ab89443), anti-XRN2 (#ab72181), anti-EXOSC10 (#ab94981), anti-METTL3 (#ab195352), anti-METTL14 (#ab220030), anti-WTAP (#ab195380), anti-HA (#ab1424), anti-FLAG (#ab125243, #ab236777), anti-XRN1 (#ab70259), and anti-GAPDH (#ab181602). The YTHDC2 and SLC7A11 protein levels were also measured by using ELISA kits from Lichen Biotech Ltd (Shanghai, China). IHC was performed using conventional protocols. IHC scores were computed through multiplying staining intensity grade (0, 1, 2, or 3 represented negative, weak-positive, moderate-positive and strong-positive, respectively) by positive rate score (0, 1, 2, 3 or 4 represented positive areas of ≤ 5%, 6–25%, 26–50%, 51–75% and ≥ 76%, respectively). The antibodies used for IHC were: anti-YTHDC2 (#ab176846), anti-SLC7A11 (#ab175186), anti-PCNA (#ab92742) and anti-4-HNE (#ab48506). For RT-qPCR, the relative mRNA and RNA fragments were quantified by SYBR (Vazyme Biotech, Nanjing, China). The primers are listed in Supplementary Table S1. For co-immunoprecipitation (co-IP), cells were washed with PBS and then lysed in Western/IP lysis buffer (Beyotime, China). Then protein lysates were incubated with 3 μg indicated Abs and protein A/G beads (Life Technologies) at 4 °C overnight. Beads were washed five times with lysis buffer, resuspended in SDS loading buffer, and subjected to IB analysis.

### 2.7 Study of m^6^A

For dot blot assay, total RNA was isolated with Trizol and mRNA was enriched using Dynabeads mRNA purification Kit (Invitrogen). mRNA was denatured by heating at 72 °C for 10min, followed by chilling on ice immediately. Then, mRNA was spotted on Biodyne Nylon Transfer Membranes (Pall, New York, NY, USA) and cross-linked by UVP for 10 min. The m^6^A level was measured using the m^6^A antibody (Synaptic Systems, #202003, Goettingen, Germany). For global m^6^A level, the quantity was measured by the m^6^A RNA methylation assay kit (Abcam, #ab185912). MeRIP-seq was performed by RiboBio (Guangzhou, China). For luciferase experiments, CDS and partial 3′UTR contain consensus m^6^A motif of *SLC7A1*1 mRNA were cloned into the pmir-GLO plasmids. For mutant reporter vectors, adenosine (A) in the m^6^A motif was replaced by a cytosine (C). The CDS, WT and Mut PCR products of SLC7A11 were synthesized by Generay Biotech Ltd (Shanghai, China). The pmir-GLO plasmids were transiently transfected into the indicated cells, and the luciferase activity was measured 24 h later using a kit from Promega (Madison, WI, USA). The primers were listed in Supplementary Table S1.

### 2.8 Promoter analysis

The human *SLC7A11* -2 K promoter was cloned into the pGL4-Basic plasmids. Transfection efficiency was normalized by co-transfection with the Renilla plasmids. The primers were listed in Supplementary Table S1.

### 2.9 RNA and protein interactions

For m^6^A immunoprecipitation qPCR (MeRIP-qPCR), total RNA was extracted. After fragmentation, RNA was incubated with anti-m^6^A antibodies for immunoprecipitation using Magna methylated RNA immune-precipitation (MeRIP) m^6^A kit (Millipore, #17–10499, Billerica, MA, USA). Enrichment of m^6^A containing mRNA was then measured using RT-qPCR assay. For RNA immunoprecipitation (RIP) experiments, a Magna RIP Kit (Millipore, #17–700) was used. Briefly, 10 cm dish cell lysates were incubated with magnetic beads loaded with 5 μg antibodies of YTHDC2 (Abcam, #ab176846) or IgG overnight at 4 °C. The remaining RNA after proteinase K digestion was purified using TRIzol and measured by RT-qPCR. The primers for MeRIP and RIP-qPCR are supplied in Supplementary Table S1. For photoactivatable ribonucleotide crosslinking and immunoprecipitation (PAR-CLIP), control cells and cells stably expressing YTHDC2^WT^-HA or YTHDC2 ^ΔYTH^-HA were cultured with 4-SU (250 μM, Sigma) for 16 h, then irradiated with 365 nm UV light for crosslinking. The protein-RNA complex was labeled with biotin using the RNA 3′end biotinylation kit (Thermo, #20160, Waltham, MA, USA). After washing three times with IP wash buffer, beads were resuspended and boiled at 95 °C for 10 min. To detect RNA-protein complexes, the samples were separated by SDS-PAGE and visualized by the chemiluminescent nucleic acid detection module (Thermo, #89880). RNA fragments were extracted by proteinase K digestion of the gel slices followed by ethanol precipitation. And the purified RNA pellets were dissolved in DEPC water and subjected to RT-qPCR detection. For in vitro RNA pull-down assay, synthesized partial *SLC7A11* 3′UTR probes with or without m^6^A modification at GGAC motif or mutate GGAC motif to CCAG were labeled by biotin (Takara, Dalian, China). About 1 × 10^7^ H1299 cells with control, YTHDC2^WT^-HA or YTHDC2 ^ΔYTH^-HA groups were washed with cold PBS and lysed in protein lysis buffer. Then incubated with 3 μg biotinylated *SLC7A11* 3′UTR probes at 4 °C overnight. The biotin-coupled RNA-protein complex was pull-downed with streptavidin magnetic beads (Life Technologies, Carlsbad, CA, USA) for another 4 h. After incubation and washing five times, the streptavidin beads were boiled and used for the IB assay.

### 2.10 Evaluation of lipid peroxidation

To ascertain the involvement of lipid peroxidation in lung tumorigenesis, the cells were stained by 5 μM fluorescent probe C11-BODIPY^581/591^ for 30 min at 37 °C followed by flow cytometry. To visualize the membrane, tissue slides were incubated with 25 μg/ml Concanavalin A-Alexa Fluor^TM^ 350 (Thermo, #C11254) following 5 μM C11-BODIPY^581/591^ staining at 37 °C for 30 min. Images were captured at emission at 580/600 nm (the non-oxidized form, red) and 490/510 nm (the oxidized form, green) and then merged to demonstrate the fraction of the oxidized C11-BODIPY^581/591^. The malondialdehyde (MDA) and 4-hydroxynonenal (4-HNE) concentration were assessed using lipid peroxidation assay kits (Abcam, #ab118970 and #ab238538). IHC was measured to detect the expression level of 4-HNE using anti-4-HNE antibodies (Abcam, #ab46545).

### 2.11 Measurement of metabolites

Metabonomics was performed by Luming Biotech Ltd (Shanghai, China). Cystine concentration were measured using an ELISA kit (Lichen Biotech). Glutamate release assay was determined using a glutamate detection kit (Solarbio, #BC1580). GSH/GSSG ratio was determined by GSH and GSSG levels measured by GSH (Solarbio, #BC1175) and GSSG (Solarbio, #BC1180) assay kits. The NAD^+^/NADH ratio was measured using the NAD^+^/NADH Assay Kit (Abcam, #ab65348) according to the manufacturer's instructions. For cystine uptake, medium was removed and replaced with cystine-free medium. L-^14^C-Cystine (0.2 μCi/mL, PerkinElmer, #NEC854010UC, Shelton, CT, USA) was added and further incubated for 30 min before it was washed and lysed. To quantify ^14^C radioactive cystine uptake, cell lysate was added into 1 ml scintillation fluid and radioactive ^14^C counts per minute (CPM) were obtained in a liquid scintillation counter (Tri-Carb 2910 TR, PerkinElmer).

### 2.12 Statistical analysis

*P* values were calculated with Student's *t*-test between two groups. One-way ANOVA and two-way ANOVA were used to compare multiple groups. To investigate the correlation between two independent groups, the *Chi-*squared test was used. Survival curves were generated using the Kaplan-Meier method and compared using the log-rank test. Data are presented as means ± SEMs from three independent experiments. *p < 0.05 and **p < 0.01 were considered statistically significant.

## 3 Results

### 3.1 Suppression of YTHDC2 is associated with tumor progression in LUAD

**Fig. 1:**
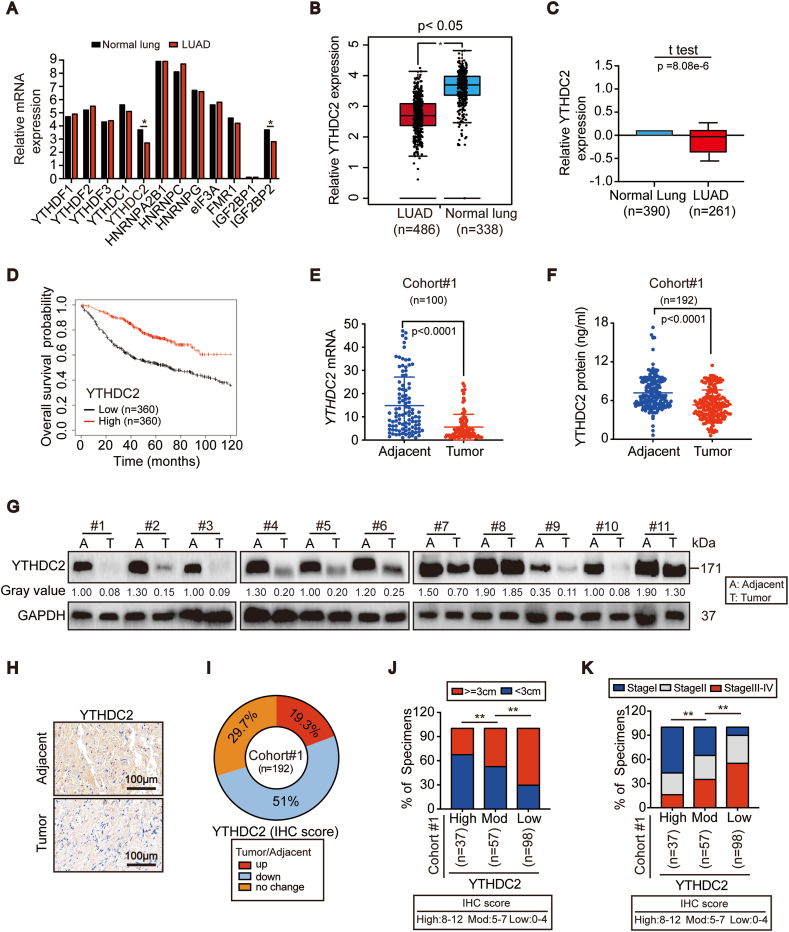
**YTHDC2 expression was suppressed in LUAD and correlated with poor clinical outcome.** (A–C) Expression level of m^6^A readers in the normal lung and LUAD tissues from GEPIA (A, B) and Oncomine (C) databases. (D) Overall survival curves based on YTHDC2 expression in LUAD were obtained from Kaplan-Meier plotter database. (E–G) YTHDC2 mRNA and protein expression in paired adjacent-tumor tissues from cohort#1, as measured by RT-qPCR, ELISA, and IB assay. (H, I) Representative IHC images of YTHDC2 in paired adjacent-tumor tissues from cohort#1 TMA, scar bar 100 μm. IHC staining was scored and analyzed, and the percentage for patients with indicated alteration of YTHDC2 is also shown. (J, K) Correlations between YTHDC2 expression and tumor diameter and between YTHDC2 expression and tumor stage. Statistical analysis was performed using Student's t tests (E, F) and *Chi*-squared test (J, K). Data are means ± SEMs, *p < 0.05, **p < 0.01.

To evaluate the expression profile of m^6^A readers in lung cancer, we analyzed known readers, including YTHDF1/2/3, YTHDC1/2, HNRNPA2B1, HNRNPC/G, eIF3A, FMR1 and IGF2BP1/2, in LUAD and lung squamous cell carcinoma (LUSC) via the GEPIA database. Among these, only YTHDC2 was downregulated in both LUAD and LUSC compared to normal lung. However, IGF2BP2 was differentially expressed in LUAD and LUSC (Fig. 1A and B, Figs. S1A and B). The Oncomine databases (Fig. 1C and S1D-G) also revealed that YTHDC2 was commonly downregulated in LUAD. Analysis of the Kaplan-Meier plot further demonstrated that lower YTHDC2 correlated with poorer overall survival in LUAD (Fig. 1D) but not in LUSC (Fig. S1C). These results led us to investigate the role of YTHDC2 in LUAD.

In cohort #1, which consisted of recruited LUAD patients in Shanghai, China, a significant downregulation of *YTHDC2* mRNA and protein was observed in tumors compared to adjacent normal tissues (Fig. 1E–G). Similarly, YTHDC2 in established LUAD cell lines was decreased compared to that in BEAS-2B cells (Fig. S1H). The tissue microarray assay (TMA) assessing YTHDC2 expression in cohort #1 demonstrated that YTHDC2 was downregulated in 51% (98/192) of LUAD patients (Fig. 1H and I). Notably, downregulation of YTHDC2 was associated with larger tumor diameters and a more advanced stage (Fig. 1J and K), indicating that YTHDC2 suppression promotes tumor progression in LUAD.

### 3.2 YTHDC2 exhibits antitumor activity via its m^6^A-reading domain

**Fig. 2:**
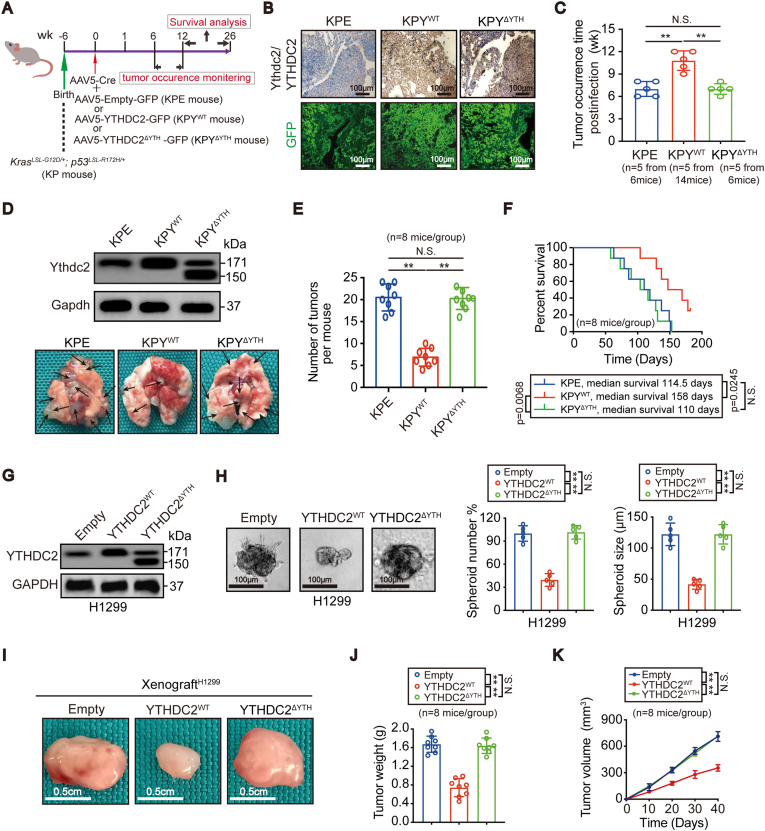
**Overexpressing YTHDC2 suppressed cell viability and tumorigenesis.** (A) Schematic presentation of the generation and monitoring of KPE, KPY^WT^ and KPY^ΔYTH^ spontaneous LUAD mouse models. (B-F) Representative GFP and IHC staining of Ythdc2/YTHDC2, tumor occurrence time, images of lungs (black arrows indicate tumors), quantification of tumors, and survival curves of KPE, KPY^WT^ and KPY^ΔYTH^ mice (log-rank test, KPE versus KPY^WT^ p = 0.0245, KPY^WT^ versus KPY^ΔYTH^ p = 0.0068, KPE versus KPY^ΔYTH^ no significant). (G) IB analysis for YTHDC2 expression in H1299 control cells, YTHDC2^WT^ or YTHDC2^ΔYTH^ overexpression cells. (H) 3D spheroid formation in H1299 control cells, YTHDC2^WT^ or YTHDC2^ΔYTH^ overexpression cells, scar bar 100 μm. Spheroids were counted at 7 days after culture for those with Φ values of greater than 30 μm, but smaller than 150 μm. (I) Representative images of xenografts formed by control cells, H1299 cells with YTHDC2^WT^ or YTHDC2^ΔYTH^ overexpression, scar bar 0.5 cm. (J, K) Tumor weights were assessed after sacrificing mice. Tumor volumes were monitored every 10 days (n = 8 per group). Statistical analysis was performed using one-way ANOVA (C, E, H and J) and two-way ANOVA (K). Data are means ± SEMs, **p < 0.01, N·S.: no significant.

Conditional activation of oncogenic Kras and inactivation of p53 in KP mice by viruses that express Cre in lung epithelial cells results in a rapid development of LUAD [[20], [21], [22]]. To explore the m^6^A reader function of YTHDC2 in vivo, we achieved pulmonary overexpression of YTHDC2 with or without the m^6^A-recognizing YTH domain and generated KP-based and GFP-tagged KPY^WT^, KPY^ΔYTH^ and control KPE mice (Fig. 2A). In addition to strong GFP signals, YTHDC2 was confirmed to be upregulated for a sustained period in tumors from KPY^WT^ and KPY^ΔYTH^ mice compared to those of KPE mice 25 wk post infection (Fig. 2B), indicating long-lasting efficiency of the AAV5 expression system. Although YTHDC2 was unable to stop lung tumorigenesis, the tumor occurrence time was delayed, the overwhelming lung tumor burden was significantly suppressed, and the survival time was longer in KPY^WT^ mice than in KPE mice and KPY^ΔYTH^ mice (Fig. 2C–F).

We further investigated whether YTHDC2 is a tumor suppressor in human LUAD cells. H1975 and H1299 cells were chosen because they exhibited the highest and lowest YTHDC2 expression among the LUAD cell lines tested, respectively (Fig. S1H). IB assays confirmed the overexpression and knockout efficiency of YTHDC2 (Fig. 2G, S2A). While the number and size of the 3D spheroids and the tumor growth of xenografts decreased following YTHDC2^WT^ overexpression in H1299 cells, these effects were not observed upon YTHDC2^ΔYTH^ overexpression (Fig. 2H–K). In contrast, knocking out YTHDC2 in H1975 cells increased spheroid formation and xenograft growth in mice (Figs. S2B–E). Notably, when YTHDC2 expression was reconstituted using YTHDC2^sg2−resistant (res)^, the positive roles of YTHDC2-sg2 in tumorigenesis were restored (Figs. S2B–E). Thus, YTHDC2 exerts a tumor suppressive function in LUAD via its m^6^A reading domain.

To exclude potential competitive interference with endogenous YTHDC2 function in our overexpression experiments, YTHDC2 activity was reconstituted by FLAG-tagged YTHDC2^sg2−res^ in YTHDC2-sg2-mediated YTHDC2 knockout H1299 cells, and its expression was adjusted to a similar level in NC-sg-treated control cells before further overexpressing HA-tagged YTHDC2^sg2−res^ or YTHDC2^△YTH (sg2−res)^ (Fig. S2F). Because YTHDC2 has the ability to interact with the 5′-3′ exoribonuclease XRN1 [23], the YTHDC2-XRN1 interaction was evaluated to assess YTHDC2 function in co-IP experiments. By assessing XRN1 levels in immunoprecipitates obtained using anti-FLAG antibodies, we observed that the YTHDC2-XRN1 interactions were not affected by HA-tagged YTHDC2^sg2−res^ or YTHDC2^△YTH (sg2−res)^ overexpression (Fig. S2G), indicating that competitive interference with endogenous YTHDC2 be ignored in our overexpression experiments. These results also explained why the significant expression of Ythdc2 in murine tumors (Fig. 2D) and YTHDC2 in H1299 cells (Fig. 2G) did not alter the transformative phenotypes when the mutant was overexpressed (Fig. 2D–K).

### 3.3 YTHDC2 inhibits cystine uptake and the downstream antioxidant program

**Fig. 3:**
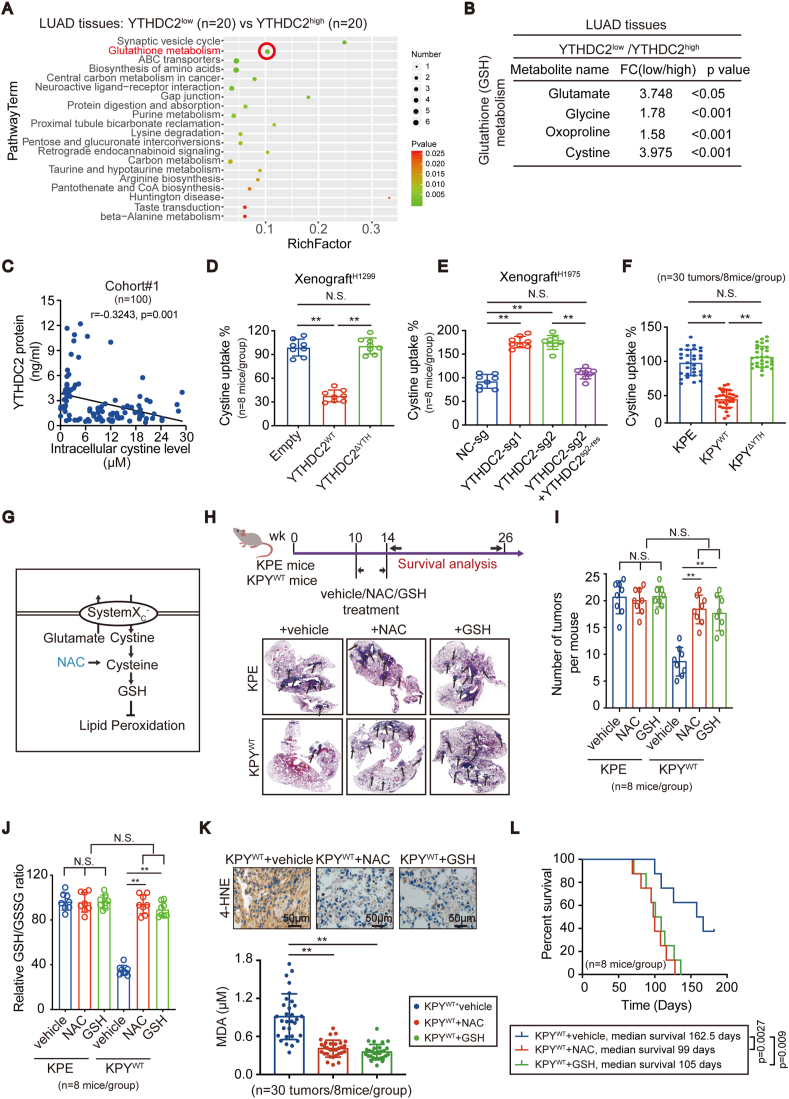
**YTHDC2 suppressed cystine uptake and downstream antioxidant program in LUAD.** (A, B) Statistics of top 20 enriched KEGG pathways, and alterations in metabolites involved in GSH metabolism, as measured by metabolomics in YTHDC2^low^ (n = 20) and YTHDC2^high^ (n = 20) LUAD tissues. (C) Correlation between intracellular cystine level and YTHDC2 protein in LUAD tissues from cohort #1 (n = 100, pearson analysis, p = 0.001). (D–F) Cystine uptake in xenografts (n = 8 per group) generated by H1299 cells with YTHDC2^WT^ or YTHDC2^ΔYTH^ overexpression, H1975 cells with or without YTHDC2 knockout and reconstitution, and tumors from KPE/KPY^WT^/KPY^ΔYTH^ mice (n = 30 tumors from 8 mice per group), as detected by L-^14^C-cystine (0.2 μCi/mL). (G) Schematic presentation of antioxidant program from cystine uptake to lipid peroxidation. (H–J) Schematic illustration of drug treatment strategy. Representative H&E staining of lungs bearing tumors (black arrows indicate tumors), quantification of tumors and relative GSH/GSSG ratio in KPE and KPY^WT^ mice (n = 8 per group) administrated with vehicle, NAC (100 mg/kg/day) or GSH (100 mg/kg/day) for 4 weeks. (K, L) 4-HNE, MDA (n = 30 tumors from 8 mice per group), and survival curves in KPY^WT^ mice (n = 8 per group) administrated with vehicle, NAC (100 mg/kg/day) or GSH (100 mg/kg/day), as determined by IHC, lipid peroxidation assays, and the Kaplan-Meier method (log-rank tests, vehicle versus NAC: p = 0.0027, vehicle versus GSH: p = 0.009), scar bar 50 μm. Statistical analysis was performed using one-way ANOVA (D-F, I-K). Data are means ± SEMs, **p < 0.01, N·S.: no significant.

Metabolic activity is typically observed in tumors with high protumorigenic properties. To investigate the impacts of YTHDC2 on metabolites, a metabolomic analysis was performed comparing LUAD specimens with low and high YTHDC2 expression (referred to as YTHDC2^low^ and YTHDC2^high^, respectively) (n = 20/each group). GSH metabolism was among the top 20 KEGG pathways identified (Fig. 3A). Among the significantly altered metabolites, a 3.975-fold increase in cystine was observed in YTHDC2^low^ tumors compared to YTHDC2^high^ tumors (Fig. 3B). Moreover, a negative correlation between YTHDC2 and intracellular cystine was observed in cohort #1 (n = 100) (Fig. 3C). These data indicate that YTHDC2 reduces intracellular cystine.

To verify whether YTHDC2 reduces cystine uptake, we cultured primary patient-derived LUAD cells with either high (pHY#1–8) or low (pLY#1–8) YTHDC2 expression levels (Fig. S3A). Extracellular cystine levels in the culture medium were lower in pLY#1–8 cells than in pHY#1–8 cells, while intracellular cystine levels were higher (Figs. S3B and C). The L-^14^C-cystine uptake assay results showed that YTHDC2, through its YTH domain, suppressed cystine uptake in xenografts generated by H1299 and H1975 cells, in LUAD formed in mice and in patient-derived LUAD cells (Fig. 3D–F, S3D). Upregulation of *CHAC1* mRNA levels has been shown to indicate impaired cystine uptake [15]. Indeed, in the present, YTHDC2 was observed to increase *CHAC1* mRNA levels (Figs. S3G and K). The cystine-glutamate antiporter system Xc^−^ imports cystine with the counter transport of glutamate. The glutamate release assay results showed that YTHDC2 suppresses glutamate release (Fig. S3H and L). These results suggest that YTHDC2 impairs cystine uptake via the inhibition of system Xc^−^. System Xc^−^ is crucial for redox balance and metabolism [24,25], and alteration of the GSH/GSSG and NAD^+^/NADH ratios in H1299 and H1975 cells by the YTH domain of YTHDC2 (Figs. S3I and J, M and N) further suggested that YTHDC2 may impair system Xc^−^ function.

Impaired system Xc^−^ function was associated with decreased GSH levels (illustrated in Fig. 3G and Ref. 15, 26). Data from patient-derived LUAD cells and tumors in mice demonstrated that GSH was indeed reduced by YTHDC2 (Figs. S3E and F). To investigate whether downstream cystine antioxidants compensate for the antitumor roles of YTHDC2, mice were administered GSH and NAC, a cysteine precursor. The tumor burden in the lung was significantly higher in KPY^WT^ mice with NAC and GSH administration than in the vehicle-treated control mice, and the levels were similar to that of the KPE mice (Fig. 3H and I). Interestingly, GSH and NAC administration only resulted in a significant increase in the GSH/GSSG ratio in the tumors from KPY^WT^ mice compared to those from KPE mice (Fig. 3J). A potential reason for this result is that YTHDC2 impairs system Xc^−^, and we proposed that the impairment of system Xc^−^ function might be critical for the facilitation of GSH and its raw materials other than cystine, such as cysteine or its precursor to enter into cell. This potential mechanism is consistent with the findings of a study describing that supplementation of GSH or NAC could effectively reverse the effects of cystine deprivation by cystine starvation or inhibiting system Xc^−^ [27].

As reported in prior studies [28,29] and shown in Fig. 3G, the lack of GSH leads to an increase in lipid peroxidation. Therefore, a fluorescent probe (C11-BODIPY^581/591^) was used to measure oxidized lipids in mouse LUAD. To specifically avoid GFP interference, we constructed KPE, KPY^WT^ and KPY^ΔYTH^ mice without a GFP tag. As shown in Fig. S3O, the fraction of oxidized C11-BODIPY^581/591^ (green) was largely increased in the tumors of KPY^WT^ mice compared to those of KPE mice; however, these levels were similar in the tumors of KPE and KPY^ΔYTH^ mice. Moreover, 4-HNE and MDA levels, which are products of lipid peroxidation, were increased in the tumors of KPY^WT^ but not in KPY^ΔYTH^ mice compared to KPE mice (Fig. S3P). Notably, NAC and GSH supplementation reduced lipid peroxidation and shortened survival in KPY^WT^ mice (Fig. 3K and L). Overall, these results indicate the m^6^A reading function of YTHDC2 is essential to reduce the cystine downstream antioxidant program.

### 3.4 YTHDC2 suppresses SLC7A11 expression

While suppressed system Xc^−^ function has been implicated in YTHDC2-impaired cystine uptake (Fig. 3), how YTHDC2 regulates system Xc^−^ remains unclear. SLC7A11, the catalytic subunit, plays a large role in maintaining system Xc^−^ activity [16,30]. Using the UALCAN database, we found that SLC7A11 was upregulated in LUAD compared to normal lung (Fig. S4A). Moreover, higher SLC7A11 led to a shorter overall survival of LUAD patients (Fig. S4B). Notably, YTHDC2 was inversely correlated with SLC7A11 in LUAD (Fig. S4C). Thus, SLC7A11 might function opposite of YTHDC2 to maintain cystine uptake.

**Fig. 4:**
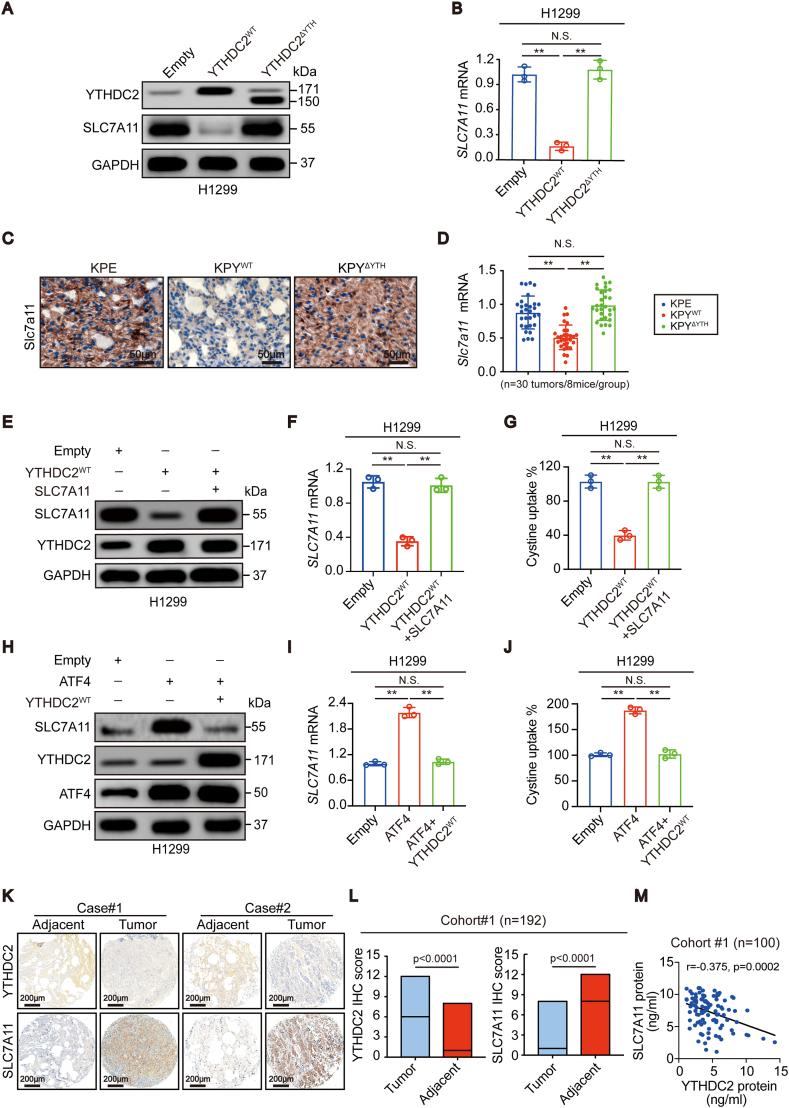
**YTHDC2 suppressed cystine uptake dependent on SLC7A11.** (A, B) SLC7A11 protein and mRNA expression in H1299 control cells, YTHDC2^WT^ and YTHDC2^ΔYTH^ overexpression cells, as determined by IB and RT-qPCR assay. (C, D) IHC and RT-qPCR assay showed Slc7a11 expression in KPE, KPY^WT^ and KPY^ΔYTH^ tumor tissues (n = 30 tumors from 8 mice per group), scar bar 50 μm. (E–G) SLC7A11 protein expression, mRNA level and cystine uptake were measured in H1299 control cells, YTHDC2^WT^ overexpression cells with or without simultaneously overexpressed SLC7A11, as detected by IB, RT-qPCR and L-^14^C-cystine (0.2 μCi/mL). (H–J) SLC7A11 protein expression, mRNA level and cystine uptake were measured in H1299 control cells, ATF4 overexpression cells with or without simultaneously overexpressed YTHDC2^WT^, as detected by IB, RT-qPCR and L-^14^C-cystine (0.2 μCi/mL). (K–M) Representative IHC images of YTHDC2 and SLC7A11 expression from two paired LUAD cases in corhort#1 TMA (K), scar bar 200 μm. Proteins were quantified by IHC scores (L). And the correlation between YTHDC2 protein and SLC7A11 protein level in cohort#1 (pearson analysis, p = 0.0002) (M). Statistical analysis was performed using one-way ANOVA (B, D, F, G, I, J) *Chi*-squared test (L). Data are means ± SEMs, **p < 0.01, N·S.: no significant.

Next, we investigated whether YTHDC2 regulates SLC7A11. The protein and mRNA levels of SLC7A11 were both negatively regulated by YTHDC2 in H1299 and H1975 cells (Fig. 4A and B, S4D and E). In mice, Slc7a11 was downregulated in tumors from KPY^WT^ mice compared to those from KPE and KPY^ΔYTH^ mice (Fig. 4C and D). In addition, the YTH domain is a prerequisite for YTHDC2 to suppress SLC7A11 expression (Fig. 4A–D).

### 3.5 YTHDC2 impairs cystine uptake via SLC7A11

Subsequently, we investigated whether YTHDC2 inhibits cystine uptake via SLC7A11. Ectopically expressing SLC7A11 completely restored the impaired cystine uptake when YTHDC2 was overexpressed in H1299 cells (Fig. 4E–G). In contrast, YTHDC2 deletion-induced cystine uptake was reversed by shRNA targeting SLC7A11 in H1975 cells (Figs. S4F–H). ATF4 and NRF2 are two critical transcription factors for *SLC7A11* transcription [31,32]. Unlike ATF4, NRF2 was unable to upregulate SLC7A11 in H1299 cells (Fig. 4H, S4I and J), which might be due to a LUAD cell context-specific effect. Notably, overexpressing YTHDC2 still antagonized the effects of ATF4 (Fig. 4H–J). Hence, YTHDC2 impairs cystine uptake dependent on SLC7A11.

The clinical relevance of YTHDC2 and SLC7A11 in cohort #1 was also investigated. YTHDC2 was downregulated, whereas SLC7A11 was upregulated in tumors compared to adjacent tissues (Fig. 4K and L, S4K). In addition, they were negatively correlated in tumors (Fig. 4M).

### 3.6 YTHDC2 accelerates *SLC7A1*1 mRNA decay

**Fig. 5:**
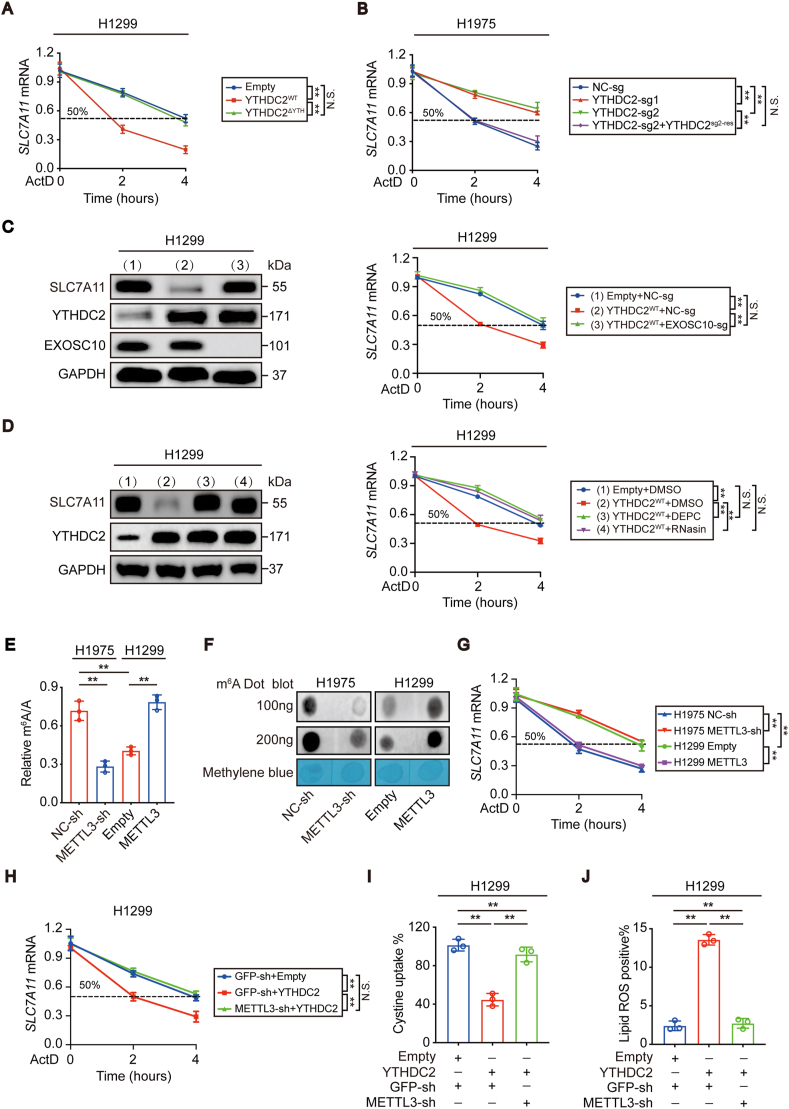
**YTHDC2 destabilized *SLC7A1*1 mRNA in an m**^**6**^**A-dependent manner.** (A, B) The decay rate of *SLC7A1*1 mRNA in control cells, H1299 cells with YTHDC2^WT^ or YTHDC2^ΔYTH^ overexpression, and H1975 cells with or without YTHDC2 knocked out or reconstitution after ActD (5 μg/mL) treatment at the indicated times. (C) SLC7A11 protein and mRNA expression in H1299 control cells and YTHDC2^WT^ overexpression cells with or without EXOSC10 knocked out. (D) SLC7A11 protein and mRNA expression in H1299 control cells and YTHDC2^WT^ overexpression cells with or without DMSO, DEPC or RNasin (5 U/μl) treatment. (E–G) The m^6^A levels and decay rate of *SLC7A1*1 mRNA were measured by m^6^A RNA methylation assay kit, m^6^A dot blot and RT-qPCR assays in control cells, H1975 cells with METTL3 knockdown, and H1299 cells with METTL3 overexpression. (H–J) The decay rate of *SLC7A1*1 mRNA, cystine uptake and Lipid ROS generation were detected in H1299 control cells, YTHDC2 overexpression cells with or without knocked METTL3 down. Statistical analysis was performed using two-way ANOVA (A-D, G, H) and one-way ANOVA (E, I, J). Data are means ± SEMs, **p < 0.01, N·S.: no significant.

The mechanisms underlying the regulation of SLC7A11 expression through YTHDC2 were further investigated. Because both the mRNA and protein levels of SLC7A11 are negatively regulated by YTHDC2 (Fig. 4 and S4), we wanted to determine whether the mRNA and protein levels of SLC7A11 were both suppressed by YTHDC2. To address this, we first investigated whether YTHDC2 regulates *SLC7A1*1 mRNA. mRNA homeostasis is maintained by the “production-degradation” balance [33,34]. The YTH domain played no role in suppressing *SLC7A11* promoter activity when YTHDC2 was overexpressed in H1299 cells (Fig. S5A). Knocking out YTHDC2 followed by reconstituting its expression in H1975 cells also voted that YTHDC2 regulates *SLC7A11* transcription (Fig. S5B). Then, we treated H1299 and H1975 cells with ActD, a pan transcription inhibitor, to examine whether YTHDC2 regulates *SLC7A1*1 mRNA stability after transcription was blocked and found that the YTH domain is essential for YTHDC2 to shorten the half-life of *SLC7A1*1 mRNA in H1299 cells (Fig. 5A). Loss of function of YTHDC2 by CRISPR/Cas9 technology followed by YTHDC2 reconstitution in H1975 cells further confirmed that YTHDC2 accelerates *SLC7A1*1 mRNA decay (Fig. 5B). XRN2 and EXOSC10 are two major exonucleases responsible for 5′-3′ and 3′-5′ exonucleolytic activity, respectively [35,36]. Deletion of EXOSC10, but not XRN2, diminished the effects of YTHDC2 on shortening the *SLC7A1*1 mRNA half-life (Fig. 5C, S5C and D), suggesting that YTHDC2 facilitates 3′-5′ *SLC7A1*1 mRNA degradation. At the pharmacological level, treating H1299 cells with DEPC and RNasin, two pan RNase inhibitors, also blocked YTHDC2 function (Fig. 5D). These findings establish a role for YTHDC2 in destabilizing *SLC7A1*1 mRNA in LUAD cells.

Subsequently, we investigated whether the protein level of SLC7A11 is simultaneously suppressed by YTHDC2 independent of its mRNA. To investigate whether YTHDC2 affects SLC7A11 translation, the CDS region of *SLC7A1*1 mRNA was cloned downstream of the firefly luciferase-encoding region of the pmirGLO plasmid. Unfortunately, the ratios between firefly luciferase and the internal Renilla luciferase were still comparable regardless of whether YTHDC2 was overexpressed or knocked out (Figs. S5E and F), suggesting that YTHDC2 is unable to regulate SLC7A11 translation. To explore whether YTHDC2 promotes SLC7A11 protein degradation, we blocked de novo generation of SLC7A11 protein by treating the indicated H1299 and H1975 cells with CHX, a protein translation inhibitor. The results showed that YTHDC2 was also unable to affect the half-life of the remaining SLC7A11 protein (Figs. S5G and H). Overall, the suppression of SLC7A11 protein by YTHDC2 might be the result of prior suppression of its mRNA.

### 3.7 *SLC7A1*1 mRNA is suppressed by YTHDC2 in an m^6^A-dependent manner

YTHDC2 regulates *SLC7A1*1 mRNA stability (Fig. 5A–D), and control of RNA decay is one of the important functions of m^6^A methylation [4,8,37]. However, m^6^A methylation of target transcripts is a prerequisite for YTHDC2 to control mRNA decay [8]. Whether the function of YTHDC2 in suppressing SLC7A11 relies on m^6^A methylation is still unknown. Among the three major writers, i.e., METTL3, METTL14, and WTAP, only a higher METTL3 expression level was found to correlate with a higher global m^6^A methylation level in H1975 cells compared to H1299 cells (Figs. S5I–K). The capacity of METTL3 to stimulate global m^6^A was also verified in H1299 and H1975 cells (Fig. 5E and F and S5L). Knocking down METTL3 in H1975 cells prolonged the half-life of *SLC7A1*1 mRNA, whereas overexpressing METTL3 in H1299 cells accelerated *SLC7A1*1 mRNA degradation (Fig. 5G). Furthermore, decreased METTL3 expression in H1299 cells blocked YTHDC2 to accelerate *SLC7A1*1 mRNA decay, impair cystine uptake and stimulate lipid reactive oxygen species (ROS) generation (Fig. 5H–J), suggesting that the role of YTHDC2 in LUAD cells is m^6^A-dependent.

### 3.8 *SLC7A1*1 mRNA is m^6^A methylated

**Fig. 6:**
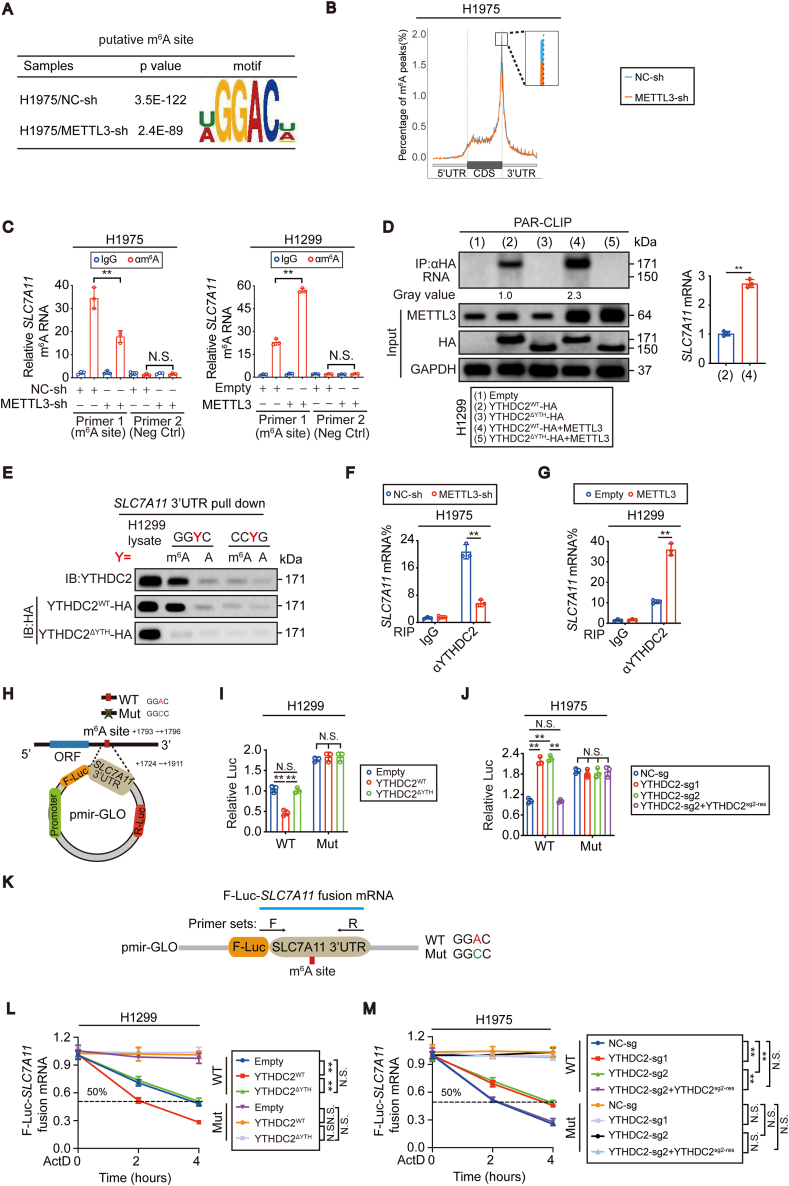
**YTHDC2 preferred to interact and destabilize m**^**6**^**A methylated *SLC7A1*1 mRNA.** (A) Predominant consensus GGAC motif was showed in H1975 cells with or without METTL3 knocked down from MeRIP-seq. (B) Density distribution of m^6^A peaks across mRNA transcripts, the percentages of m^6^A peaks were determined. (C) MeRIP-qPCR analysis of m^6^A levels at the indicated sites within *SLC7A1*1 mRNA in control cells and H1975 cells with METTL3 knockdown, and H1299 cells with METTL3 overexpressed. (D) PAR-CLIP assay of RNA pulled down by HA-tagged YTHDC2^WT^ or YTHDC2^ΔYTH^ in H1299 cells with or without overexpressing METTL3. RNA labeled with biotin at 3′ end was visualized by the chemiluminescent nucleic acid detection module. *SLC7A1*1 mRNA levels in the pulled down products were also verified by RT-qPCR. (E) *In vitro* partial *SLC7A11* 3′UTR probe pulldown assay in H1299 control cells, HA tagged YTHDC2^WT^ and YTHDC2^ΔYTH^ overexpression cells. GGYC (Y = m^6^A or A), CCYG (Y = m^6^A or A). (F, G) RIP-qPCR analysis showing the binding between YTHDC2 and *SLC7A1*1 mRNA in H1975 and H1299 cells under indicated treatment. (H–J) Schematic generation strategy for the pmir-Glo luciferase reporters containing WT and Mut (GGAC to GGCC) *SLC7A11* 3′-UTR. Luciferase activities from the indicated pmir-Glo vector were measured in H1299 and H1975 cells with or without YTHDC2 overexpression or knockout. (K) Schematic presentation of the primer sets designed to detect WT or Mut F-Luc-*SLC7A11* fusion mRNA. (L, M) The decay curve of exogenous F-Luc-*SLC7A11* fusion mRNA in indicated groups. The F-Luc-*SLC7A11* fusion mRNA level were normalized to that of internal *Renilla luciferase* mRNA. Statistical analysis was performed using Student's t tests (C, D, F, G), one-way ANOVA (I, J) and two-way ANOVA (L, M). Data are means ± SEMs, **p < 0.01, N·S.: no significant.

To reveal the potential m^6^A methylation sites in *SLC7A1*1 mRNA, MeRIP-seq was performed before and after METTL3 knockdown in H1975 cells. The GGAC motif was highly enriched within m^6^A sites in H1975 cells with or without knocking down METTL3 (Fig. 6A). m^6^A peaks were especially abundant in the 3′ untranslated region (3′UTR) just near the stop codons of mRNAs (Fig. 6B), and *SLC7A1*1 mRNA was no exception (Fig. S6A). MeRIP-seq revealed a putative m^6^A motif located within the 3′UTR (+1793~+1796 relative to the transcription start site (TSS) of the *SLC7A11* gene) (Fig. S6A). The m^6^A enrichment around this site was correlated with the m^6^A methylation level (Fig. 5E and F, Fig. S6A). To further confirm the m^6^A-dependent modification of *SLC7A1*1 mRNA, MeRIP-qPCR was performed. m^6^A modification in *SLC7A1*1 mRNA was significantly reduced specifically around the putative m^6^A site after METTL3 was knocked down in H1975 cells. In contrast, the opposite outcome was observed when METTL3 was overexpressed in H1299 cells (Fig. 6C). Thus, we provided evidence that *SLC7A1*1 mRNA can be m^6^A methylated in LUAD cells.

### 3.9 YTHDC2 preferentially binds to m^6^A-methylated *SLC7A1*1 mRNA

Although YTHDC2 functions as an m^6^A reader, whether YTHDC2 preferentially binds to and recognizes m^6^A-methylated *SLC7A1*1 mRNA is unknown. To this end, a PAR-CLIP experiment to assess whether the *SLC7A1*1 mRNA-YTHDC2 interaction depends on m^6^A methylation was first performed. The results demonstrated that increasing the m^6^A methylation level by overexpressing METTL3 in H1299 cells facilitated YTHDC2 binding with *SLC7A1*1 mRNA (Fig. 6D). In contrast, the *SLC7A1*1 mRNA-YTHDC2 interaction was remarkably suppressed once METTL3 was knocked down in H1975 cells (Fig. S6B). Regardless of whether METTL3 was overexpressed or knocked down, deletion of the YTH domain blocked the *SLC7A1*1 mRNA-YTHDC2 interaction (Fig. 6D and S6B). Next, we performed an in vitro RNA pulldown assay using synthesized partial *SLC7A11* 3′UTR probes and incubated them with lysates from H1299 cells expressing HA-tagged YTHDC2^WT^ and YTHDC2^ΔYTH^. YTHDC2^WT^, but not YTHDC2^ΔYTH^, bound preferentially to the 3′UTR of *SLC7A1*1 mRNA containing an m^6^A compared to the one with an unmethylated adenosine (Fig. 6E). Additionally, YTHDC2 is prone to bind the m^6^A consensus motif GGAC, which is the same as that located within the putative m^6^A site, because a robust reduction in the *SLC7A11* 3′UTR-YTHDC2 interaction was observed when GGAC was replaced by a random CCAG motif (Fig. 6E). Finally, a RIP-qPCR assay was performed to evaluate whether m^6^A methylation modulates the intracellular *SLC7A1*1 mRNA-YTHDC2 interaction. Indeed, higher global m^6^A methylation caused a stronger *SLC7A1*1 mRNA-YTHDC2 interaction in H1975 cells than in H1299 cells (Fig. S5J and K, S6C). From the gain- and loss-of-function experiments of METTL3 in H1299 and H1975 cells, we also concluded that the intracellular *SLC7A1*1 mRNA-YTHDC2 interaction is determined by the m^6^A level (Fig. 6F and G). These data suggest that YTHDC2 preferentially binds to m^6^A-methylated *SLC7A1*1 mRNA.

### 3.10 YTHDC2 destabilizes *SLC7A1*1 mRNA in an m^6^A-dependent manner

Then, we examined whether YTHDC2 destabilizes *SLC7A1*1 mRNA via the putative m^6^A site. We cloned the wild-type (WT) *SLC7A11* 3′UTR (+1724 ~ +1911 relative to the TSS) and mutant (Mut) 3′UTR (GGAC to GGCC) downstream of the firefly luciferase (F-Luc) encoding region in the pmirGLO plasmid (Fig. 6H). In this experiment, firefly luciferase activities correlated with mRNA stability. The results suggested that only overexpression of YTHDC2^WT^ but not YTHDC2^ΔYTH^ reduced *SLC7A1*1 mRNA stability in H1299 cells and that knocking out YTHDC2 could increase its stability in H1975 cells. However, compared to the those of the WT 3′UTR, these effects were diminished for the Mut reporter (Fig. 6I and J). Similar results were obtained after examining the RNA stability of the exogenous *F-Luc-SLC7A11* fusion mRNA expressed by the pmirGLO plasmid (Fig. 6K-M). Overall, the m^6^A site at the 3′UTR is essential for YTHDC2 to destabilize *SLC7A1*1 mRNA.

### 3.11 LUAD with YTHDC2 suppression is sensitive to system X_C_^−^-targeting therapy

**Fig. 7:**
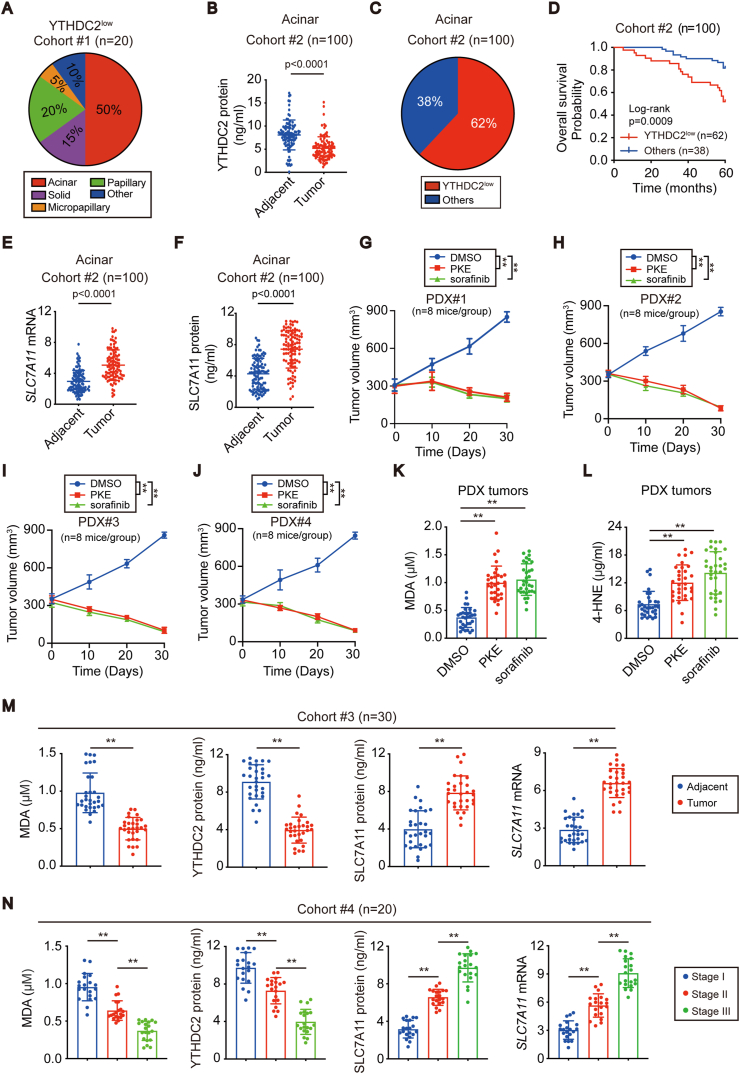
**YTHDC2 suppression was sensitive to system X**_**C**_^**-**^**-targeting therapy and associated with LUAD progression.** (A) Percentages of the indicated subtypes in cohort#1 LUAD with the YTHDC2^low^ expression pattern. (B) YTHDC2 protein levels in paired acinar LUAD tissues (cohort #2), as measured by ELISA. (C) Percentages of LUAD with the YTHDC2^low^ expression pattern in acinar LUAD (cohort #2). (D) Overall survival in acinar LUAD (cohort #2) with or without the YTHDC2^low^ expression pattern (log-rank test, p = 0.0009). (E, F) *SLC7A1*1 mRNA and protein levels were measured in paired acinar LUAD tissues (cohort #2) using RT-qPCR and ELISA assay. (G–J) Tumor volume was calculated in PDX mice models administrated with DMSO, PKE (20 mg/kg/day) or sorafenib (80 mg/kg/day) at the indicated time points. (K, L) MDA and 4-HNE levels were detected using lipid peroxidation assay kit in PDX tumors. (M, N) MDA, YTHDC2 protein levels, SLC7A11 protein and mRNA levels were measured by lipid peroxidation assay, ELISA, and RT-qPCR in cohorts #3 and #4. Statistical analysis was performed using Student's t tests (B, E, F, M), two-way ANOVA (G–J) and one-way ANOVA (K, L, N). Data are means ± SEMs, **p < 0.01.

To evaluate the importance of YTHDC2 suppression in LUAD, twenty YTHDC2^low^ LUADs were randomly selected from cohort #1, and 50% of them belonged to the acinar subtype (Fig. 7A). To understand the prevalence of YTHDC2^low^ in the acinar subtype, another cohort #2 was recruited specific for the acinar subtype. In cohort #2 (n = 100), YTHDC2 was downregulated in the tumor compared to the adjacent tissues, and 62% (62/100) of acinar tissue were classified as YTHDC2^low^ (Fig. 7B and C). Overall survival was much shorter among acinar LUAD patients with YTHDC2^low^ compared to those without this expression pattern (Fig. 7D), further demonstrating that YTHDC2 suppression might be especially important for tumor progression of acinar LUAD.

YTHDC2 suppresses SLC7A11 (Fig. 4, S4); therefore, we speculated that SLC7A11 is simultaneously upregulated in acinar LUAD. Indeed, this is true in cohort #2 (Fig. 7E and F). Recently, utilizing ystem X_C_^−^ inhibitors, such as erastin and its derivatives, has been proven to be an attractive strategy to treat cancer [16,38]. We wondered whether piperazine erastin (PKE), a stable in vivo derivative of erastin, has a potential therapeutic effect on acinar LUADs. In addition, sorafenib, an FDA-approved multikinase inhibitor and a potent system X_C_^−^ inhibitor, was examined in parallel. The PDX mouse model is valuable to evaluate treatment sensitivity in patients [39]. Hence, PDX models that originated from 4 distinct acinar LUAD patients were administered PKE and sorafenib. No significant changes in histopathology and expression of YTHDC2 and SLC7A11 were observed due to mouse passage (Fig. S7A). Compared to that in the DMSO-treated control, significant tumor regression was observed when PKE and sorafenib were administered (Fig. 7G–J, S7B). In addition, administrating mice with PKE and sorafenib also caused a significant increase in MDA and 4-HNE in tumors (Fig. 7K and L), suggesting that induced lipid peroxidation might be the result of inhibiting tumorigenesis in acinar LUADs.

Finally, we recruited another two cohorts to verify our conclusion. While cohort #3 LUAD patients were recruited from North China, cohort #4 was also recruited from Shanghai, China, but during a time frame distinct from that of cohort #1. Similar to the observations for cohort #1, MDA and YTHDC2 were suppressed, while both the mRNA and protein levels of SLC7A11 were elevated in the tumor compared to the adjacent tissues in cohort #3 (n = 30) (Fig. 7M), confirming that suppression of YTHDC2 in LUAD prevents lipid peroxidation by upregulating SLC7A11. In cohort #4 (n = 20/each stage), MDA and YTHDC2 were negatively associated, while SLC7A11 was positively associated with the stage (Fig. 7N), demonstrating that SLC7A11 elevation might be the key for YTHDC2 suppression to promote LUAD progression.

## 4 Discussion

Recently, as a hot topic in the field of epigenetic regulation, RNA m^6^A modification has been shown to participate in multiple cellular processes, such as the maturation and degradation of mRNA and protein translation [7,40]. The functions of m^6^A modification have been studied in multiple cancers, such as leukemia, liver, colon and breast cancer [6,[41], [42], [43]]. High levels of the m^6^A writer METTL3 significantly promote liver and breast cancer progression [44,45]. By contrast, low levels of the m^6^A erasers FTO and ALKBH5 are linked with poor renal carcinoma prognosis [46]. In addition, m^6^A readers such as the YTH and IGF2BPs families play contradictory roles and act as either tumor promoters or suppressors [7,47,48]. Nevertheless, these studies highlight that the interplay in the WER system is essential to regulate cancer initiation and progression. However, how m^6^A works through its readers and what their targets are in LUAD remain unclear. Thus, from the perspective of the reader, the present study demonstrates that YTHDC2 has antitumor activity in LUAD and that *SLC7A1*1 mRNA is its direct target.

Our current understanding of the YTH proteins, which consist of YTHDF1, YTHDF2, YTHDF3 and YTHDC1, suggests that this m^6^A reader family is critical for tumorigenesis [7,[48], [49], [50]]. However, the role of YTHDC2 remains unclear. To address this, we investigated the function of YTHDC2 in LUAD tumorigenesis. Our results demonstrated that YTHDC2 suppression is frequent and is associated with tumor progression in LUAD. Furthermore, we showed that YTHDC2 impairs system X_C_^−^ activity and cystine uptake through downregulation of SLC7A11. m^6^A methylation by METTL3 is a prerequisite for YTHDC2 to accelerate *SLC7A1*1 mRNA decay. Hence, upregulation of SLC7A11 in LUAD is a result of YTHDC2 suppression. Because cystine is indispensable for the downstream antioxidant program [26,51], suppression of YTHDC2 thus promotes tumorigenesis by protecting LUAD cells from oxidative damage.

Metabolic active is one of the major characteristics that distinguishes tumor cells from normal cells [52,53]. Although ROS are physiologically important, excessive ROS are harmful for tumor cells [54]. Accumulated lipid peroxidation is one such oxidative damage [55]. The antioxidant system, including the system X_C_^−^/GSH axis, thus far has shown extreme importance to prevent excessive lipid peroxidation [26,56]. The activity of system X_C_^−^ is often positively correlated with the expression of its catalytic subunit SLC7A11 [15,57]. SLC7A11 can be transcriptionally regulated by a series of transcription factors [18,58,59]. It can also be regulated at the protein level [16,17,60]. Our results further showed that YTHDC2 is associated with lipid peroxidation and that it regulates *SLC7A1*1 mRNA and protein expression in a manner dependent on posttranscriptional regulation, in which *SLC7A1*1 mRNA is destabilized in an m^6^A- and YTHDC2-dependent manner. Although prior studies have demonstrated that upregulation of *SLC7A1*1 mRNA is a negative feedback to system X_C_^−^ inhibition [14,19], overexpression of YTHDC2 only causes a downregulation of *SLC7A1*1 mRNA. This does not necessarily mean that system X_C_^−^ is not inhibited because *SLC7A1*1 mRNA has a short half-life of less than 4 h; therefore, according to our model, YTHDC2-mediated reduction of system X_C_^−^ activity might not be a slow process.

Acinar subtype, the most common and heterogeneous type of LUAD, can be further divided into prognostically significant subsets [61,62]. Here, we suggest that the acinar subtype can be further classified according to YTHDC2 expression because acinar with low YTHDC2 expression is associated with poor survival. Fortunately, acinar LUAD is still sensitive to the system X_C_^−^ inhibitors PKE and sorafenib. This might be because SLC7A11 is the potential target of both agents [16,63]. In other words, elevated SLC7A11 in acinar LUAD somehow increases the opportunity for PKE and sorafenib to exert their antitumor effects. Abnormal activation of oncoproteins often occurs in tumors. The application of small molecular inhibitors targeting these proteins in clinical oncology has shown great potential to improve patient outcomes [[64], [65], [66]]. Our preclinical model thus provides another example that molecularly selected patients might benefit from small molecule targeted therapy.

**Fig. 8:**
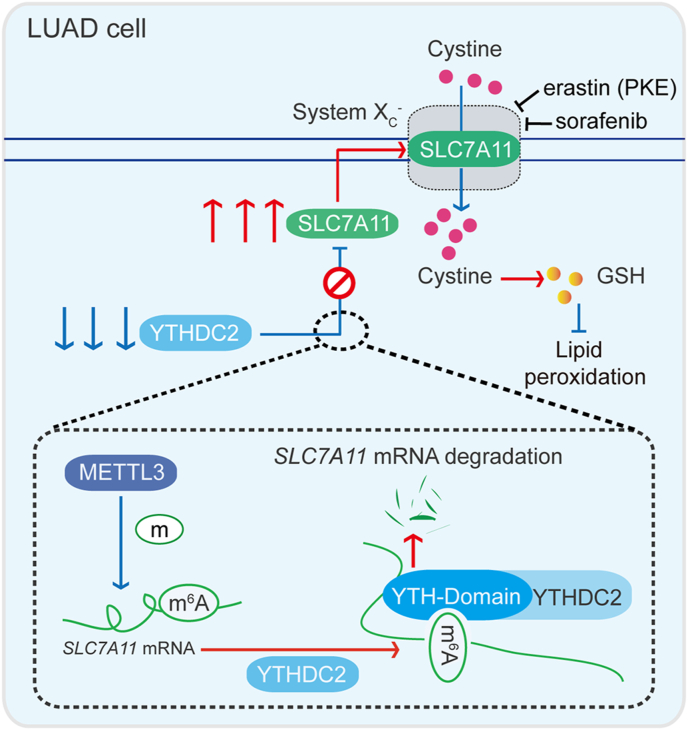
**Model of the study.** The diagram reveals an m^6^A-dependent YTHDC2 regulation of *SLC7A1*1 mRNA to modulate cystine uptake in LUAD cells. In our model, YTHDC2 destabilizes *SLC7A1*1 mRNA via its m^6^A-reading YTH domain. METTL3-mediated m^6^A methylation of *SLC7A1*1 mRNA at its 3′UTR is prerequisite for this process. YTHDC2 is frequently suppressed in LUAD; thereby its anti-tumor activity to impair cystine uptake and downstream antioxidant program is blocked. This model may explain a possible mechanism underlying tumorigenesis in LUAD.

In conclusion, this study emphasizes the importance of the m^6^A reader YTHDC2 in LUAD. System X_C_^−^ activity is induced to promote tumorigenesis possibly by suppressing YTHDC2 (Fig. 8). Furthermore, our results suggest that molecular subtyping based on YTHDC2 expression might be helpful to predict prognosis in LUAD. Inhibitors targeting system X_C_^−^ might be helpful to treat LUAD patients with poor prognosis. Thus, this study provides valuable insights regarding tumorigenesis, molecular subtyping, and drug sensitivity in LUAD.

## Data availability

The high-throughput MeRIP sequencing data have been deposited in the Gene Expression Omnibus (GEO) database under the accession number GSE147171. All data supporting the findings of this study are included in the manuscript and are available from the corresponding author upon request.

## Author contributions

JY Wang, YC Yu and LT Du conceived of the study and JY Wang carried out its design. LF Ma, TX Chen and X Zhang performed the experiments. YY Miao, XT Tian and KK Yu collected clinical samples. LF Ma, X Xu, YJ Niu, SS Guo, CC Zhang, SY Qiu, YX Qiao and WT Fang analyzed the data. LF Ma and JY Wang wrote the paper. YC Yu and LT Du revised the paper. All authors read and approved the final manuscript.

## Declaration of competing interest

The authors declare that they have no known competing financial interests or personal relationships that could have appeared to influence the work reported in this paper.
