# Supplementary material for: The m6A reader YTHDC2 inhibits lung adenocarcinoma tumorigenesis by suppressing SLC7A11-dependent antioxidant function
Source: Redox Biol. 2020 Nov 18;38:101801. doi: 10.1016/j.redox.2020.101801 (PMC7691619; doi:10.1016/j.redox.2020.101801)
Supplement: Multimedia component 1 [file mmc1.docx]

**Supplementary Table S1**

| **The sequence of shRNA, sgRNA, and cloning primers** |
| --- |
| h-sh-METTL3: GCTGCACTTCAGACGAATTAT |
| h-sg1-YTHDC2: GCTGGAGCGCTTCCGATACG |
| h-sg2-YTHDC2: GGCGGCGGCCCCTCGCCTTG |
| h-sg-XRN2: GTTGTGCAGTCGTACGTTGA |
| h-sg-EXOSC10: GTGGCAGTCACCAAGGCATC |
| h-sg-NRF2: GCGACGGAAAGAGTATGAGC |
| h-YTHDC2^WT^-HA-F: CCCAAGCTTATGTACCCATACGACGTCCCAGACTACGCTTCCAGGCCGAGCAGCGTCTCCC |
| h-YTHDC2^WT^-HA-R: CGGGGTACCCTAATCAGTTGTGTTTTTTTCTCCC |
| h-YTHDC2^ΔYTH^-HA-R: CGGGGTACCCTAAGGCATGTTTGGTCTTGGCGAA |
| h-PGL4-SLC7A11-2000bp-F: GTACGGTACCTAAATGTCGTCATGTTTTGC |
| h-PGL4-SLC7A11-2000bp-R: GATCAAGCTTCGTAGCTGGAGAGTTATTTC |
| h-pmir-Glo-SLC7A11-F:GATCGCTAGCGAGAAAATAACCAGAACATTA |
| h-pmir-Glo-SLC7A11-R:GACTCTCGAGAAAAATAACTGACTCCTTTTG |
|  |
| **The primer sequence of qPCR, MeRIP and RIP-qPCR** |
| h-YTHDC2-qPCR-F:CAACTCCTAGTAATGAACGGAAGC |
| h-YTHDC2-qPCR-R:TTAAATACTCCTCCTAGTCCAGC |
| h-GAPDH-qPCR-F:ATCATCCCTGCCTCTACTGG |
| h-GAPDH-qPCR-R:GTCAGGTCCACCACTGACAC |
| h-SLC7A11-qPCR-F:TCCCTCTATTCGGACCCATTTA |
| h-SLC7A11-qPCR-R:TTCTTCTGGTACAACTTCCAGT |
| h-CHAC1-qPCR-F:ATTGCCACGCAGATCCTGGCCTG |
| h-CHAC1-qPCR-R:CATGAAGTCTGCCAGACGCAGCA |
| h-F-Luc-SLC7A11 fusion-F: CTAGTTGTTTAAACGAGCT |
| h- F-Luc-SLC7A11 fusion-R: GCAGGTCGACTCTAGACTC |
| m-Gapdh-qPCR-F:AGGTCGGTGTGAACGGATTTG |
| m-Gapdh-qPCR-R:GGGGTCGTTGATGGCAACA |
| m-Slc7a11-qPCR-F:GGCACCGTCATCGGATCAG |
| m-Slc7a11-qPCR-R:CTCCACAGGCAGACCAGAAAA |
| h-SLC7A11-RIP-qPCR-F: GATAGGGCGGCAGCAGCA |
| h-SLC7A11-RIP-qPCR-R: CTTTCAACTTTGGTGTCT |
| h-SLC7A11-MeIP-qPCR-F1: GGCTTCGTCATCACTCTG |
| h-SLC7A11-MeIP-qPCR-R1: CTCCTTTTGTTTATCACC |
| h-SLC7A11-MeIP-qPCR-F2: GATAGGGCGGCAGCAGCA |
| h-SLC7A11-MeIP-qPCR-R2: CTTTCAACTTTGGTGTCT |
|  |

**TableS1. The sequences of shRNA, sgRNA and primers for cloning, qPCR, MeRIP and RIP-qPCR.**
