# Supplementary material for: The m6A reader YTHDC2 inhibits lung adenocarcinoma tumorigenesis by suppressing SLC7A11-dependent antioxidant function
Source: Redox Biol. 2020 Nov 18;38:101801. doi: 10.1016/j.redox.2020.101801 (PMC7691619; doi:10.1016/j.redox.2020.101801)
Supplement: Multimedia component 2 [file mmc2.docx]

**Supplementary Figures S1–S7**

**FigureS1
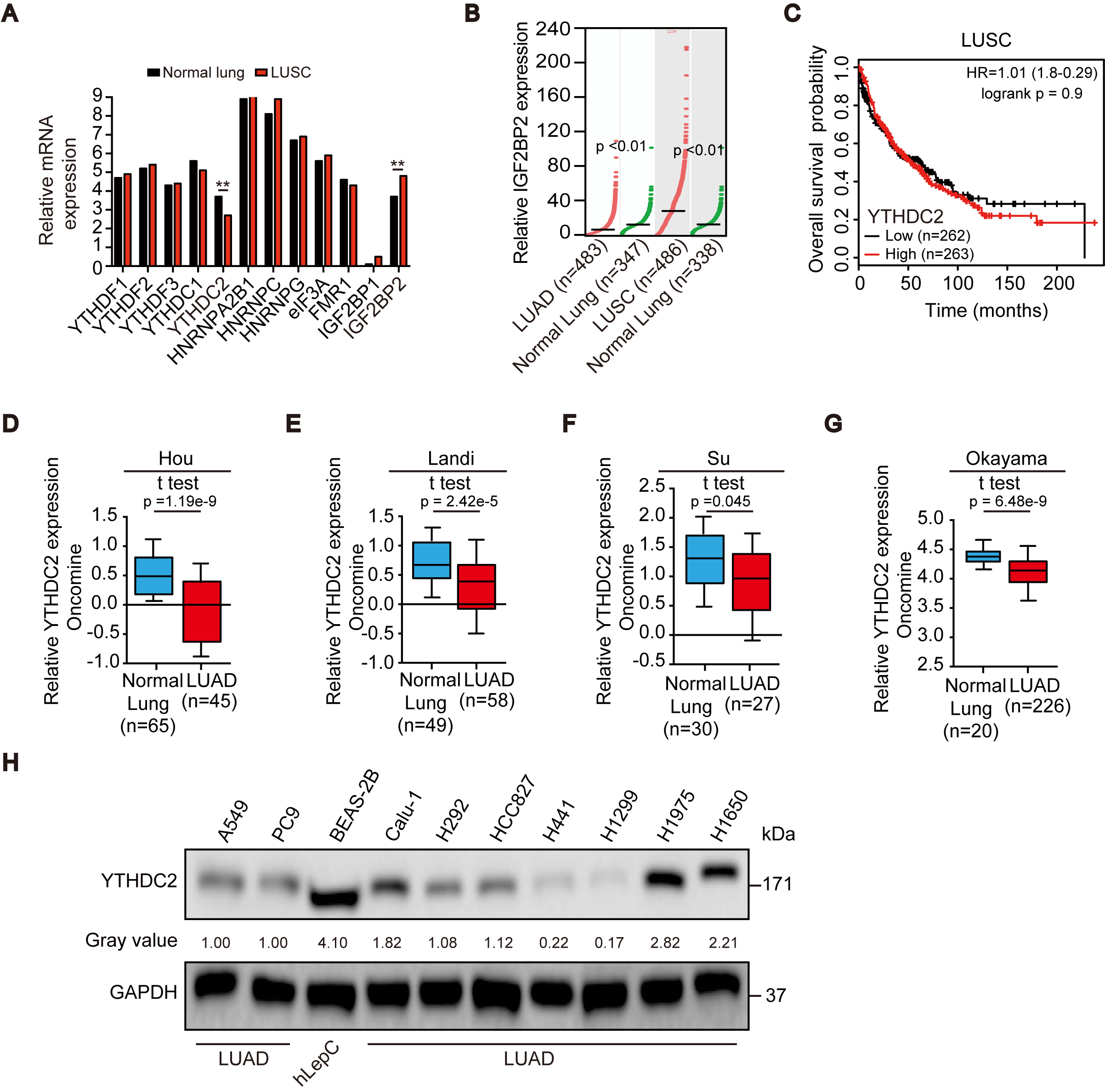
**

**Figure S1. YTHDC2 was downregulated in LUAD tissues and cell lines.** (A) Expression level of m^6^A readers in the normal lung and LUSC tissues from GEPIA database. (B) The expression level of IGF2BP2 in normal lung, LUAD and LUSC tissues, as extracted from GEPIA database. (C) Overall survival curves based on YTHDC2 expression in LUSC were obtained from Kaplan-Meier plotter database. (D-G) Relative *YTHDC2* mRNA expression level in normal lung and LUAD tissues from Oncomine database. (H) IB analysis of the YTHDC2 proteins in hLepC and LUAD cell lines.

**FigureS2**


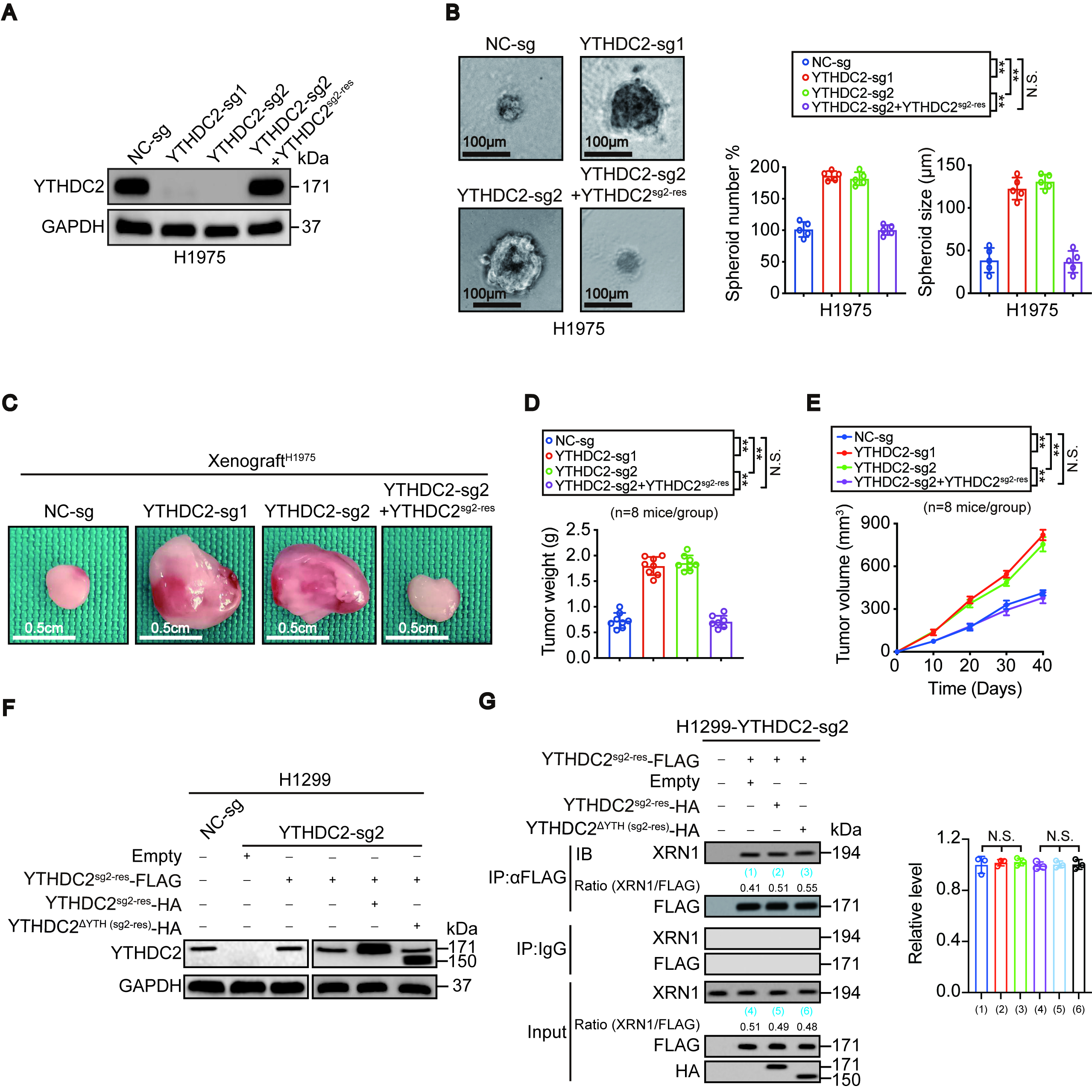


**Figure S2. Silencing of YTHDC2 promoted cell viability and tumor growth.** (A) YTHDC2 knocked out efficiency was measured by IB analysis in H1975 cells. (B) 3D spheroid formation in indicated H1975 cells, scar bar 100μm. Spheroids were counted at 7 days after culture for those with Φ values of greater than 30 μm, but smaller than 150 μm. (C) Representative images of xenografts formed by H1975 cells with YTHDC2 knocked out with or without reconstitution by an sg2-resistant YTHDC2, scar bar 0.5cm. (D, E) Tumor weights were assessed after sacrificing mice and tumor volumes were monitored every 10 days (n=8 per group). (F) YTHDC2 expression level in indicated groups. (G) Co-IP of FLAG-tagged YTHDC2^sg2-res^ and XRN1 in H1299 cells with or without HA-tagged YTHDC2 ^sg2-res^ or YTHDC2^ΔYTH (sg2-res)^ overexpression, and the relative levels of XRN1 (normalized to FLAG) in indicated group were also graphed. Statistical analysis was performed using one-way ANOVA (B, D and G) and two-way ANOVA (E). Data are means ± SEMs, **p < 0.01, N.S.: no significant.

**FigureS3**

**
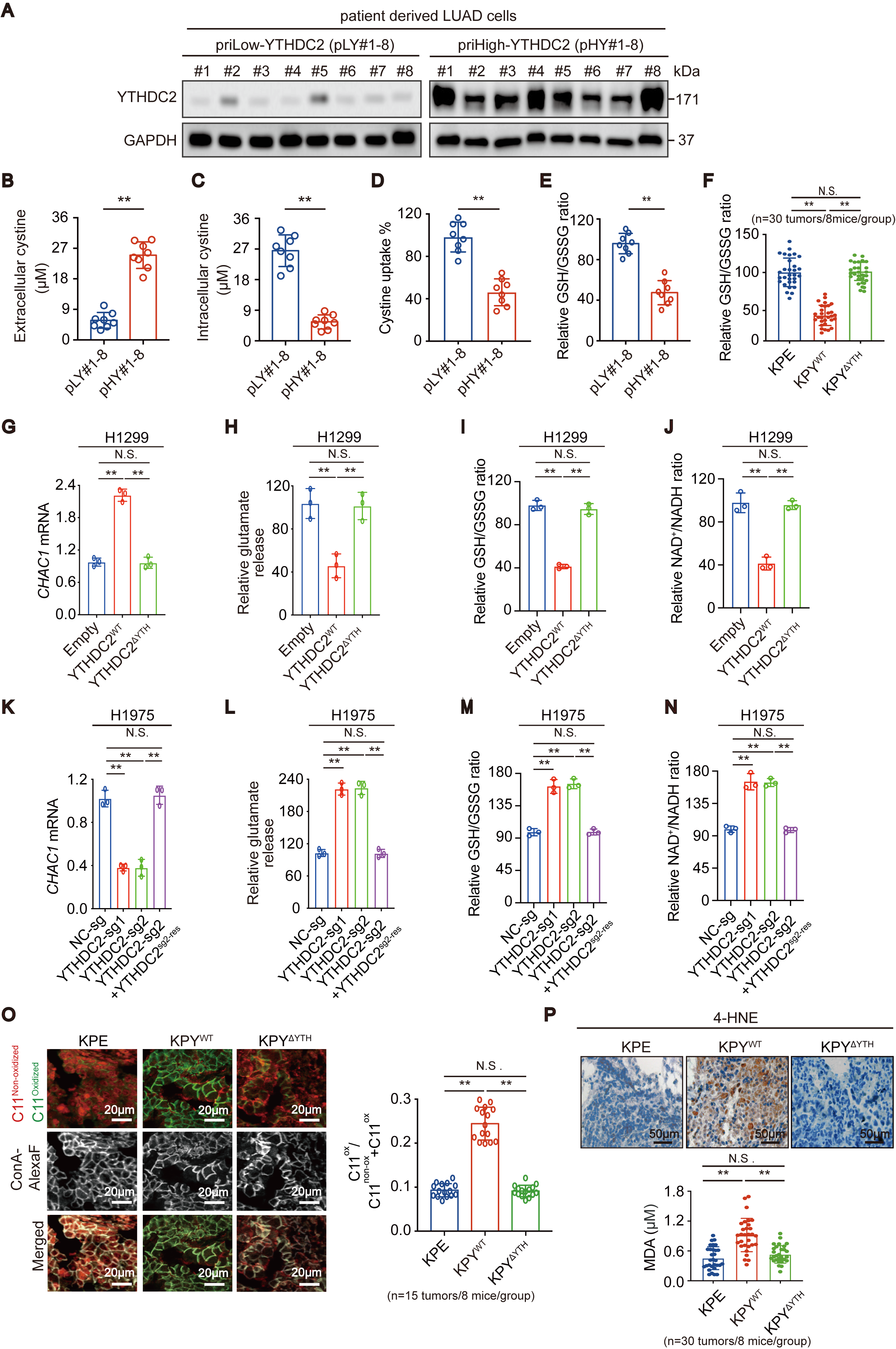
**

**Figure S3. YTHDC2 suppressed cystine uptake and downstream antioxidant program in LUAD.** (A) YTHDC2 expression level in patient-derived pLY#1-8 and pHY#1-8 cells. (B-D) Extracellular media cystine, intracellular cystine and cystine uptake in pLY#1-8 and pHY#1-8 cells, as measured by ELISA and L-^14^C-cystine (0.2 μCi/mL). (E, F) Relative GSH/GSSG ratio in pLY#1-8 and pHY#1-8 cells and tumors from KPE, KPY^WT^ and KPY^ΔYTH^ mice, as determined by GSH and GSSG detection assay kits. (G-N) *CHAC1* mRNA levels, relative glutamate release, relative GSH/GSSG ratio and NAD^+^/NADH ratio were measured by RT-qPCR, glutamate detection assays, GSH, GSSG and NAD^+^/NADH detection assay in H1299 control cells, YTHDC2^WT^ and YTHDC2^ΔYTH^ overexpression cells, and H1975 cells with YTHDC2 knocked out with or without reconstitution. (O) Representative confocal images of tumor tissues co-stained with C11-BODIPY^581/591^ and conA-AlexaF in the LUAD from KPE, KPY^WT^, and KPY^ΔYTH^ mice (n=15 tumors from 8 mice per group), scar bar 20μm. The ratio of oxidized C11 in total C11 was quantified using Image J software. (P) 4-HNE and MDA were measured by IHC and lipid peroxidation assays in KPE, KPY^WT^, and KPY^ΔYTH^ tumor tissues (n=30 tumors from 8 mice per group), scar bar 50μm. Statistical analysis was performed using Student’s t tests (B-E) and one-way ANOVA (F-P). Data are means ± SEMs, **p < 0.01, N.S.: no significant.

**FigureS4**

**
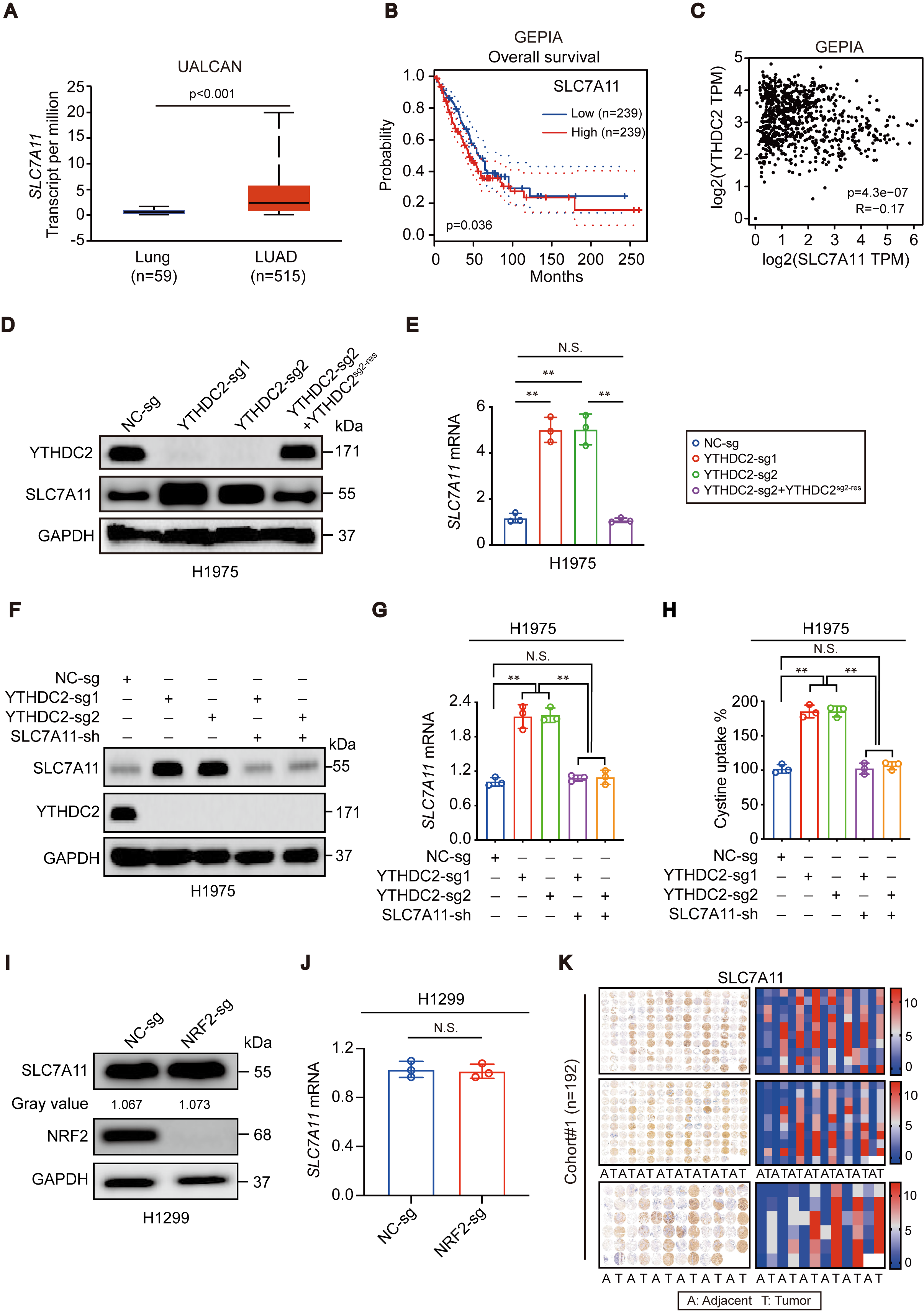
**

**Figure S4. YTHDC2 negatively regulated SLC7A11 and cystine uptake.** (A) Expression level of SLC7A11 were evaluated in normal lung and LUAD tissues via UALCAN. (B) Overall survival for patients with different SLC7A11 expression levels, as evaluated using GEPIA database. (C) Correlations between YTHDC2 and SLC7A11 were evaluated via GEPIA database. (D, E) SLC7A11 protein and mRNA expression in YTHDC2 knocked out H1975 cells with or without reconstitution, as determined by IB and RT-qPCR assay. (F-H) SLC7A11 protein expression, mRNA level and cystine uptake were measured in YTHDC2 knocked out H1975 cells with or without simultaneously knocked down SLC7A11, as detected by IB, RT-qPCR and L-^14^C-cystine (0.2 μCi/mL). (I, J) IB and RT-qPCR analysis showed SLC7A11 protein and mRNA expression in H1299 cells with or without NRF2 knocked out. (K) TMA IHC images of SLC7A11 and heat map of SLC7A11 IHC scores in corhort#1. Statistical analysis was performed using one-way ANOVA (E, G, H) and Student’s t tests (J). Data are means ± SEMs, **p < 0.01, N.S.: no significant.

**FigureS5**

**
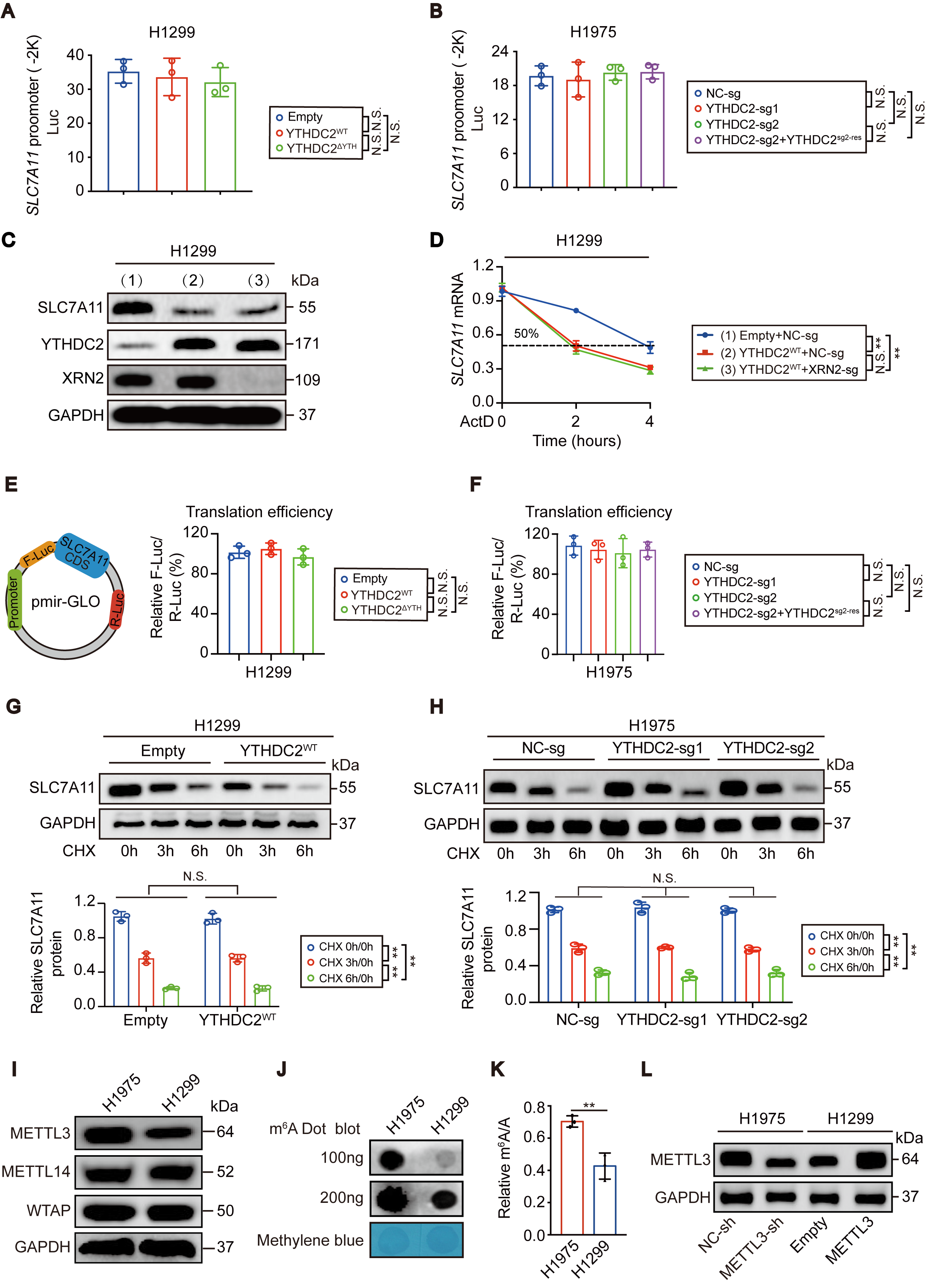
**

**Figure S5. YTHDC2 regulated SLC7A11 independent of transcription, protein translation and degradation.** (A, B) The promoter activity from the -2K region relative to the TSS of the human *SLC7A11* gene were measured in H1299 control cells, YTHDC2^WT^ and YTHDC2^ΔYTH^ overexpression cells, and H1975 cells with or without YTHDC2 knockout or reconstitution. (C, D) SLC7A11 protein expression and mRNA decay curve were measured in H1299 control cells, YTHDC2^WT^ overexpression cells with or without XRN2 knocked out using IB and RT-qPCR assay. (E, F) H1299 and H1975 cells, as indicated, were transfected with pmiR-GLO plasmids containing SLC7A11 CDS region for 24h, and the translation efficiency of SLC7A11 was illustrated as the relative ratios between F-luc and R-luc. (G, H) CHX (10 μg/mL) chase experiments for SLC7A11 protein in H1299 and H1975 cells under indicated treatment. (I) Expression levels of m^6^A writers, as measured by IB. (J, K) m^6^A levels of poly(A) RNAs were measured using an m^6^A dot blot assay and m^6^A RNA methylation quantification assay kit. (L) METTL3 knocked down and overexpressed efficiency in H1975 and H1299 cells. Statistical analysis was performed using one-way ANOVA (A, B, E-H), two-way ANOVA (D) and Student’s t tests (K). Data are means ± SEMs, **p < 0.01, N.S.: no significant.

**FigureS6**

**
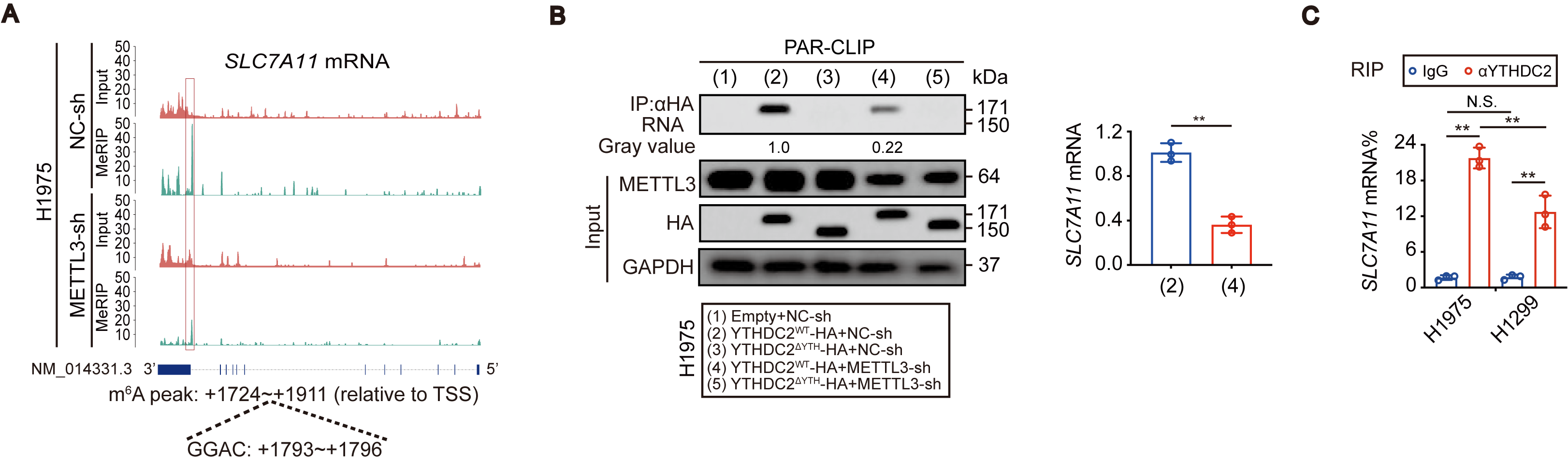
**

**Figure S6. m^6^A methylation determined YTHDC2-*SLC7A11* mRNA interaction.** (A) The m^6^A abundances within *SLC7A11* mRNA, as revealed by MeRIP-seq. (B) PAR-CLIP assay in HA-tagged YTHDC2^WT^ or YTHDC2^ΔYTH^ expressed H1975 cells with or without METTL3 knocked down. *SLC7A11* mRNA levels in the pulled down products were also evaluated by RT-qPCR. (C) RIP-qPCR analysis showing the binding between YTHDC2 and *SLC7A11* mRNA in H1975 and H1299 cells. Statistical analysis was performed using Student’s t test (B) and one-way ANOVA (C). Data are means ± SEMs, **p < 0.01, N.S.: no significant.

**FigureS7**

**
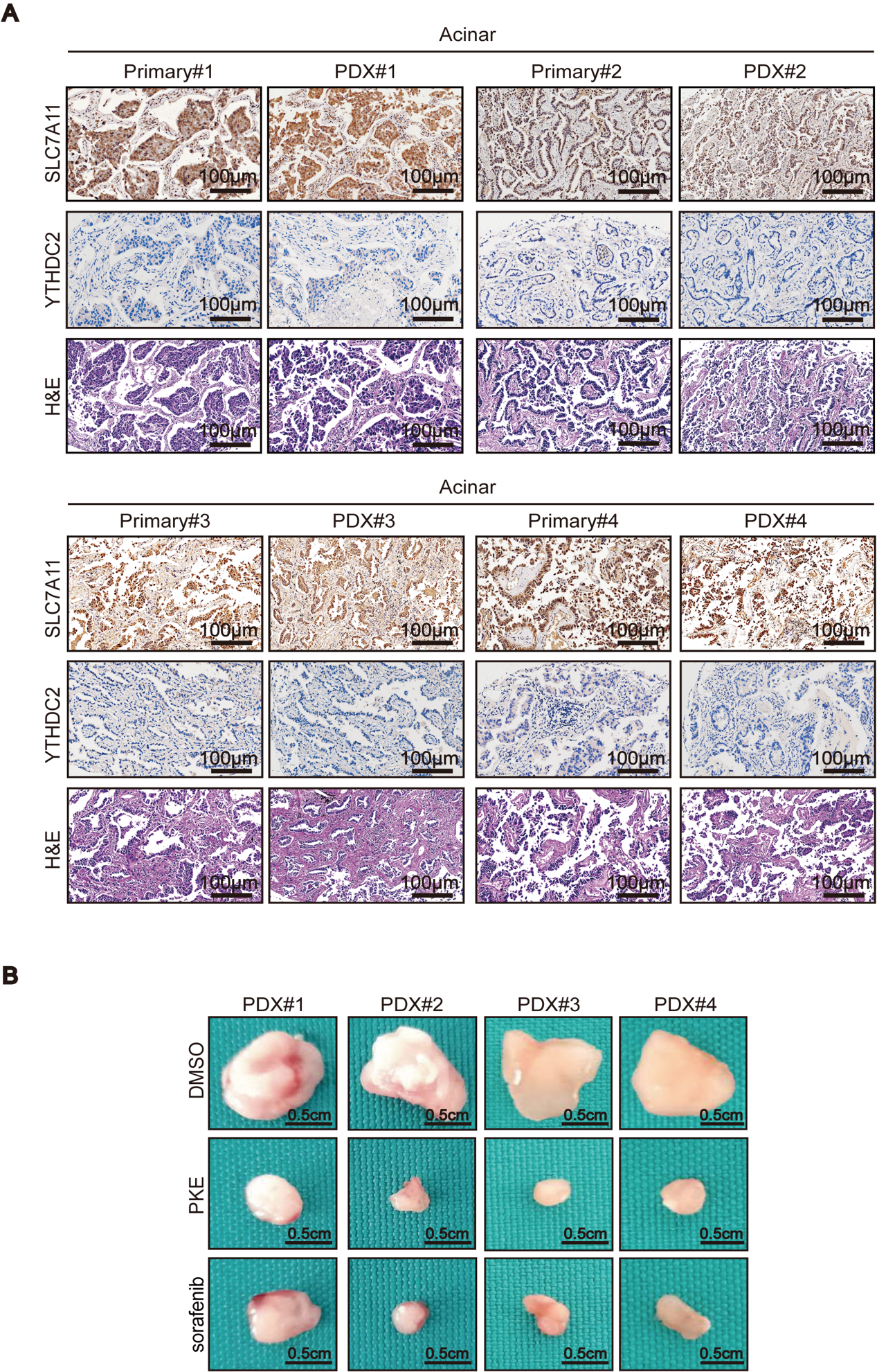
**

**Figure S7. Figure related to the Figure 7.** (A) Representative images of H&E and IHC staining by anti-YTHDC2 and anti-SLC7A11 antibodies in PDXs and their corresponding primary LUADs, scar bar 100μm. (B) Representative images of tumors from the PDX mouse models treated with DMSO, PKE (20 mg/kg/day), or sorafenib (80 mg/kg/day). Scar bar 0.5cm.
